# Sublittoral Macrobenthic Communities of Storfjord (Eastern Svalbard) and Factors Influencing Their Distribution and Structure

**DOI:** 10.3390/ani15091261

**Published:** 2025-04-29

**Authors:** Lyudmila V. Pavlova, Alexander G. Dvoretsky, Alexander A. Frolov, Olga L. Zimina, Olga Yu. Evseeva, Dinara R. Dikaeva, Zinaida Yu. Rumyantseva, Ninel N. Panteleeva, Evgeniy A. Garbul

**Affiliations:** Murmansk Marine Biological Institute of the Russian Academy of Sciences (MMBI RAS), 183038 Murmansk, Russia

**Keywords:** benthic community, environmental drivers, Barents Sea, Svalbard, Storfjord

## Abstract

Seafloor communities along the eastern Svalbard coast remain poorly studied. To address this gap, we sampled benthic organisms on the soft sediments of Storfjord in 2017 and 2019, a large fjord predominantly influenced by cold Arctic waters, to study the local fauna and identify the key environmental drivers shaping community structure. In total, 314 taxa were recorded, with an increase in abundance (from 3923 to 8977 ind. m^−2^, mean 6090 ind. m^−2^) and a decline in biomass (ranging from 265 to 104 g m^−2^, mean 188 g m^−2^) toward the outer part of the fjord. However, no clear spatial trends were observed for alpha diversity (approximately 100 species per 0.3 m^2^) or the Shannon index (mean 3 per station). The primary factors influencing benthic abundance were the duration of the ice-free period (IFP) and the degree of siltation (DS), both of which are proxies for trophic conditions. The prevailing taxa displayed a high tolerance to temperature fluctuations and seasonal variability in nutrient inputs. Benthic biomass showed a negative relationship with IFP, DS, and water depth, but it was positively correlated with the proportion of fine-grained sediment. The *Yoldia hyperborea* community (mean abundance: 3700 ind. m^−2^, mean biomass: 227 g m^−2^) was associated with Arctic waters characterized by higher inorganic suspension loads. In contrast, areas with reduced or weaker sedimentation were dominated by the communities of *Maldane sarsi* (6212 ind m^−2^, 226 g m^−2^) and *Maldane sarsi* + Nemertini g.sp. (5568 ind m^−2^, 165 g m^−2^). The *Spiochaetopterus typicus* community (7824 ind m^−2^, 139 g m^−2^) was observed in areas under moderate influence of Atlantic waters, characterized by low sedimentation rates and increased fresh detritus flux.

## Simple Summary

We sampled benthic fauna from the soft sediments of Storfjord, an eastern Svalbard fjord that has remained relatively unstudied. We identified a diverse array of taxa and observed that their abundance tended to increase while biomass decreased as we moved toward the fjord’s outer regions. The key factors influencing the abundance of benthic organisms appeared to be the duration of the ice-free period and the distance to the shoreline (the level of siltation), which reflect the overall trophic conditions. The main taxa exhibited resilience to fluctuations in temperature. We identified distinct benthic communities that demonstrated varied responses to different sediment conditions, with some communities occupying areas with higher sediment loads while others exhibit resilience in environments with reduced sedimentation. Our study highlights the dynamic nature of benthic habitats in the Arctic, suggesting that changes in climate and sedimentary conditions may influence community composition in the future.

## 1. Introduction

The Barents Sea, encompassing a vast area of 1.4 million km^2^, constitutes approximately 10% of the total area of the Arctic Ocean. Despite its relatively shallow average depth of 230 m [1], it is globally acknowledged as one of the largest continental shelves. This region plays a crucial role in high-latitude water circulation, functioning as a primary conduit for aquatic and thermal exchange between the Arctic Ocean and other global oceans. This dynamic connectivity significantly influences the mechanisms of thermal transport within both the oceanic and atmospheric systems [2]. High levels of primary production, driven by the interaction between cold Arctic water and warm Atlantic waters, support high biodiversity of local communities [3] and abundant populations of commercially important fish and shellfish species such as Atlantic cod, capelin, haddock, redfish, Greenland halibut, red king crab, snow crab, and northern shrimp [4,5]. In the past decade, commercial stocks of cod have fluctuated between 1.6 and 2 million t, while haddock stocks ranged from 175,000 to 315,000 t, saithe from 450,000 to 600,000 t, and capelin from 300,000 to 400,000 t [6]. The average landings for these species were 788,000 t for cod, 217,000 t for haddock, 163,000 t for saithe, and 123,000 t for capelin, respectively. For red king crab, the commercial biomass was reported to be between 52,000 and 199,000 t, with an average annual landing of approximately 9000 t. In contrast, snow crab exhibited a commercial biomass ranging from 275,000 to 640,000 t, with average annual landings of about 10,000 t [7,8,9,10]. In addition, the region holds considerable potential for aquaculture and recreational fishing, particularly in the coastal zones of the Kola Peninsula [11,12,13] and the Norwegian fjords [14,15].

The fjords and bays of the Barents Sea constitute high-latitude marine ecosystems that have recently attracted increasing research interest due to these ecosystems’ pronounced vulnerability to climatic fluctuations and the growing influence of human activities, including fishing, tourism, mining, and oil and gas exploration [16]. Benthic communities may be vulnerable to the consequences of anthropogenic impacts, such as waste dumping, eutrophication, and oil spills. Arctic ecosystems—characterized by low temperatures, seasonal ice cover, the prolonged polar night, a relatively short period of organic matter production, and inputs from terrestrial and glacial runoff—are unique in their pronounced seasonal cycles of productivity and ecosystem functioning.

The marine environment surrounding the Svalbard Archipelago, located in the northern Barents Sea, represents a transitional zone. It is distinguished by the dominance of warm and saline Atlantic Waters in the southern and western regions and the prevalence of colder, less saline Arctic Waters in the northern and eastern areas [17]. In recent decades, the inflow of the Atlantic water mass into the Arctic domain has become more pronounced [18,19,20,21], reflecting the ongoing process of Atlantification [22]. The western shelf of Svalbard and its adjacent fjords have experienced increasingly frequent intrusions of warmer Atlantic waters, particularly since 2011 [23]. Meanwhile, the northern shelf and shelf break of Svalbard have transitioned from relatively stable conditions—characterized by substantial ice cover, subzero temperatures in the upper water layers, and shallow mixing layer depths—to a new state typified by open water, persistently shallow water temperatures above 0 °C, and significant interannual variability in mixing layer depths and ocean-to-atmosphere heat fluxes [24]. In the northeastern fjords of the archipelago, a substantial reduction in winter sea-ice cover has been observed post-2010, following a period of abundant sea ice in the early 2000s. This trend is strongly correlated with rising surface air temperatures [25]. These ongoing transformations driven by Atlantification and pronounced warming not only alter hydrographic conditions but also trigger significant structural and functional changes in local ecosystems.

The structure and function of benthic communities are shaped by environmental drivers and faunal interactions operating across various temporal and spatial scales [26,27]. These communities, predominantly composed of sessile and long-lived organisms (polychaete worms, arthropods, mollusks, and echinoderms), are widely recognized as valuable indicators of environmental change due to their ability to integrate and reflect process variability over time [3,28,29,30]. Consequently, variations in benthic community structure are considered particularly sensitive markers of climate-driven changes [31,32] as well as other environmental disturbances, including chemical pollution [33,34], eutrophication [35,36], and biological invasions [4,37]. Fjords, characterized by distinct gradients in environmental conditions [38], are regarded as ideal locations for investigating the response of benthic communities to environmental changes, including those arising from climate variability [39,40,41,42,43,44,45,46].

Svalbard fjords, located at the confluence of the cold Arctic and warm Atlantic water masses [47], have been the focus of extensive research efforts examining benthic communities in relation to environmental drivers [40,42,48,49,50,51,52,53,54,55,56,57,58,59,60,61,62,63]. However, the majority of these studies have focused on the fjords along the west coast of Svalbard, targeting readily accessible areas such as Isfjord, Hornsund, Bellsund, Kongsfjord, and smaller adjacent fjords. In contrast, the benthic communities along the less accessible northern Svalbard coast remain comparatively understudied [44,64]. Furthermore, investigations into the impact of warming on Svalbard’s benthos remain limited, despite some efforts to study this aspect [31,44,60,65,66].

The eastern coast of Svalbard, characterized by its inaccessibility and the scarcity of available data, represents a significant gap in our understanding of benthic fauna diversity and distribution. This region, influenced by Arctic waters and marked by a prolonged period of seasonal ice cover, includes Storfjord, located in the southeastern part of the archipelago. Storfjord is largely unaffected by the heat transport associated with the West Spitsbergen Current, and its local temperature conditions are more severe and stable compared to the fjords along western Svalbard [67,68]. Nevertheless, the general trend of warming across the Svalbard archipelago [69] has intensified glacial melting in Storfjord. Despite these ongoing changes, the effects of warming on the benthic fauna in this area remain unexplored due to the absence of long-term, consistent time-series datasets.

Benthic research in Storfjord can be traced back to 1925 when a dredging survey was conducted at 13 stations by Soviet scientists [70]. However, subsequent investigations employing box-corer, SCUBA-diving, sledge, and trawl techniques have been sporadic and limited in scope, with only a few studies conducted in the area since then [71,72,73]. As a result, there is a lack of contemporary data on the state of benthic communities in Storfjord, especially during this period of rapid Arctic warming.

To bridge this knowledge gap, two benthic surveys were carried out in 2017 and 2019. The primary objectives of these surveys were to delineate spatial patterns in the diversity and abundance of benthic fauna in this region and to evaluate the influence of environmental factors on the composition and distribution of local benthic communities.

## 2. Materials and Methods

### 2.1. Study Area

**Figure 1:**
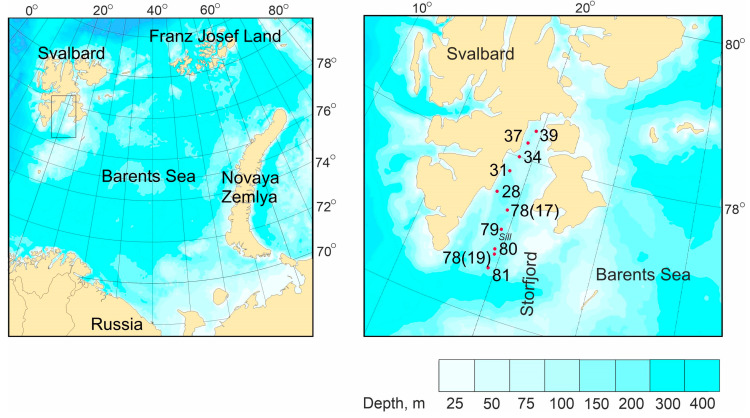
Map of the study area.

Storfjord, a large semi-enclosed bay with a depth of nearly 190 m and a length of approximately 190 km, is located southeast in the Svalbard Archipelago, bordered by the islands of Spitsbergen, Barentsøya, and Edgeøya. The fjord is defined by a 120 m deep sill at approximately 77° N to the south, a shallow bank referred to as Storfjordbanken in the southeast, and a submarine ridge in the southwest (Figure 1).

Water mass exchanges below 70 m are primarily controlled by the seafloor topography, particularly through a channel situated between 19° and 20°30′ E, which connects the sill to the trough of Storfjordrenna. The fjord predominantly receives cold and fresh Arctic Water. Warm and saline Atlantic Water enters Storfjordrenna in a cyclonic manner [74]. The local polar front, located along the slope of Storfjordrenna [75], demarcates the boundary between Arctic Water and Atlantic Water, leading to rapid water mass transformations within Storfjord that occur on a short renewal period of approximately two months [74]. The water masses in Storfjord are influenced by meltwater from numerous glaciers along the western coastline, surface inflows advected during the summer via a cyclonic coastal current, and brine-enriched bottom waters formed during winter. Brine water, generated through sea ice formation (winter water mass), is a distinctive feature of Storfjord; it is characterized by negative temperatures, high salinity, and minimal inter-annual temperature variability [76]. Importantly, Storfjord contributes approximately 5–10% of the total brine production on Arctic shelves [77,78].

Seasonal ice cover in Storfjord forms earlier and persists longer compared to the fjords of West Svalbard, significantly influencing the hydrography and ecosystem dynamics. The ice melt during spring and summer stratifies the water column, promoting abundant phytoplankton blooms along the ice edge [79]. In summer, the water column is typically divided into three distinct layers: a freshened surface layer extending to a depth of ~40 m, an intermediate layer of Arctic Water, and a brine-enriched layer usually situated below the sill depth [80,81]. The western and northern coasts of Storfjord are characterized by the presence of numerous tidewater glaciers, which are predominantly concentrated at the head of the fjord [82,83]. During the summer months, extensive terrestrial meltwater runoff strongly influences the entire coastal zone of the fjord, shaping its physical and biological properties. Storfjord exhibits some of the highest sedimentation rates, organic matter content in bottom sediments, and sedimentary burial rates across the Svalbard coastal region. The bottom sediments primarily consist of sandy and clayey silts [84,85], with a high organic carbon content, exceeding 2%, derived predominantly from terrestrial sources [86,87].

### 2.2. Sample Collection and Analysis

Sampling of macrozoobenthos was conducted during research cruises of the R/V *Dalnie Zelentsy* at four stations in July 2017 and six stations in June 2019 (Figure 1). The sampled area encompassed deep-water basins along two distinct transects. Transect I, located closer to the western coast of Storfjord, comprised relatively shallow waters. In contrast, Transect II spanned deeper waters along the central and open part of the basin. Stations 78(19), 80, and 81 were situated in the outer region of the fjord, with station 79 located within the sill area. The remaining stations were positioned further inside the fjord, covering inner basin areas.

At each station, three replicate benthic samples were collected aboard the research vessel at depths between 83 and 218 m using a van Veen grab, which has a sampling area of 0.1 m^2^. Near-bottom water temperature and salinity measurements at each sampling site were obtained using an SBE 19plus V2 CTD probe (Sea-Bird Electronics, Bellevue, WA, USA). The water conductivity data were measured by a sensor with an accuracy of 0.0005 S m^−1^ and a resolution of 0.00005 S m^−1^; the water temperature was measured by a thermometer with an accuracy of 0.005 °C and a resolution of 0.0001 °C; the water pressure was measured by a strain gauge sensor with an accuracy of 0.1% of full scale and a resolution of 0.002% of full scale. Water masses in the bottom layer were classified following the framework established by Skogseth et al. [76]: brine-enriched water (T < −1.5 °C, S > 34.8), polar front water (T = −0.5–2.0 °C, S = 34.8–35.0), and modified water (T > 0 °C, S > 34.8). Sediment characteristics were assessed visually during sampling based on parameters such as color, consistency, and grain size [88]. Sampling was performed on a polar day in daylight, and sediment characteristics were determined by the same person. The color of the surface sediments varied from light brown, gray-brown to brown, and the clay consistency ranged from soft to viscous. Substrates were classified as follows: clay (particle size < 0.005 mm), silt (0.005–0.05 mm), sandy silt (0.05–0.1 mm), sand (0.1–1.0 mm), gravel (1–10 mm), pebble (10–100 mm), stone (>100 mm), and shell (bivalve shell fragments). Additional data on surface sediment composition were derived from sedimentological analyses conducted at the same stations in 2019 and further augmented by earlier research findings [85].

The degree of terrestrial runoff influence (or degree of siltation) was evaluated based on the distance from each station to the nearest shoreline. Additionally, the duration of open-water conditions served as an indicator of seafloor organic input, given the strong influence of seasonal sea ice cover on the primary production and sedimentation of marine organic matter [89]. Information on ice conditions for the region was obtained from the Arctic and Antarctic Research Institute website (https://data.aari.ru/odata/d0004.php, accessed 18 November 2024). Specifically, the duration of the ice-free period (IFP) was calculated for each station to assess the spatial dynamics of ice cover. We summarized the months of the year when each station was ice-free. The data were averaged over the five years preceding each sampling year to reduce the effects of strong interannual cycling.

Macrozoobenthic samples were processed following standard protocols. Initially, the collected samples were rinsed through a 0.5 mm sieve to separate benthic organisms from sediments. The retained specimens were preserved in a 4% neutral-buffered formalin solution. In the laboratory, these samples were systematically re-washed, transferred to 75% ethanol for further preservation, and subsequently identified to the lowest taxonomic level possible using the most recent nomenclature provided by the World Register of Marine Species (http://marinespecies.org, accessed 20 November 2024). For each sample, organisms were enumerated and their wet weight measured to a precision of 0.0001 g. Specific weighing protocols varied across taxa: mollusks were weighed with their shells intact, while polychaetes were weighed either with their secreted tubes (for tube-dwelling polychaetes) or without tubes in the case of encrusting tube-dwelling species.

For each station, the abundance (individuals per square meter, ind. m^−2^) and biomass (grams per square meter, g m^−2^) were averaged across the three replicate samples. To provide additional insights, the mean individual body mass of benthic organisms was calculated. To assess the variability of benthic abundance and biomass, the coefficient of variation (CV) was computed for each station as the ratio of the standard deviation to the mean abundance or biomass, expressed as a percentage. Lastly, the frequency of occurrence (FO, %) for each taxon was determined across all collected samples to identify the relative distribution and prevalence of macrozoobenthic species within the sampled stations.

To quantitatively assess diversity at each station, a set of indices was employed. These included species richness (defined as the mean number of species per sample), and alpha diversity (measured as the number of taxa per 0.3 m^2^). Indices that take into account both species richness and evenness were also calculated, including the Shannon diversity index (H′) and Pielou’s evenness index (J′). Additionally, the difference in evenness index (D_E_), as proposed by Denisenko et al. [90], was used to evaluate ecological stress levels at the sampling sites. The total expected number of species (Chao2 index) was also calculated to estimate the number of species present in the whole area.

The *D_E_* index, which serves as an indicator of the ecological condition at a given station, was determined using the following formula:

$$D_{E}=\frac{J_{A}-J_{B}}{\mathrm{lg}S}$$

where *J_A_* and *J_B_* represent Shannon diversity indices derived from abundance and biomass, respectively, and *S* denotes the total number of species in the sample. The *D_E_* index yields values ranging from −1 (indicative of no ecological stress) to +1 (indicative of very strong ecological stress), with 0 representing a threshold between unstressed and stressed states.

To further investigate the contribution of individual taxa to the benthic community’s energy flow, their respiration (metabolic) rates were calculated using the formula proposed by Golikov et al. [91]:

$$R=cN^{0.25}\cdot B^{0.75}$$

where *N* is the abundance (ind. m^−2^), *B* is the biomass of the taxon (kJ m^−2^), and *c* is the specific coefficient showing the intensity of metabolism (J h^−2^). We used the following coefficients for particular groups: Hydrozoa (0.59), Actinaria (0.42), Polychaeta Sedentaria (2.10), Polychaeta Errantia (3.10), Amphipoda (2.89), Isopoda (2.89), Ostracoda (1.51), Bivalvia (2.10), Mytilidae (3.35), Gastropoda (1.76), Astartidae (0.84), Tellinidae (1.38), Gastropoda (1.76), Bryozoa (0.59), Echinodermata (0.67), and Tunicata (0.42). The biomass was converted to an energy equivalent (energy density) using the specific calorie coefficient k (kJ g^−2^ ww): Hydrozoa (1.26), Actinaria (1.26), Polychaeta Sedentaria (1.88), Polychaeta Errantia (3.56), Amphipoda (3.18), Isopoda (3.18), Ostracoda (1.47), Bivalvia (1.59), Mytilidae (1.88), Astartidae (1.21), Tellinidae (1.67), Gastropoda (1.47), Bryozoa (1.26), Echinodermata (1.47), and Tunicata (1.05) [91].

### 2.3. Statistical Analysis

To identify trends in environmental characteristics across the study region, non-metric multidimensional scaling (nMDS) was performed. This analysis utilized log-transformed environmental variables, including depth, distance to shore, near-bottom water temperature and salinity, sedimentological parameters (pelite and aleurite fractions, and the fraction of particles smaller than 0.05 mm), and duration of open-water periods. For stations where data were incomplete, missing values were estimated using the following relationships:

y = 49.429e^−0.014x^ (R^2^ = 0.862) for pellite,

y = 0.4135x + 42.302 (R^2^ = 0.590) for aleurite,

y = −0.3141x + 91.821 (R^2^ = 0.8131) for particles < 0.05 mm,

where x represents the distance to the shore (in kilometers). Organic carbon and organic matter content in bottom sediments were excluded from this analysis, as prior studies indicated a poorly defined spatial gradient in these parameters within the region [84,85].

To evaluate spatial patterns in benthic community structure, cluster analysis was conducted using the Bray–Curtis similarity matrix based on benthic respiration (energy flow) rates (R). The clustering utilized group average linkage as the classification method. Prior to analysis, the data were log-transformed to minimize the impact of extreme values, and species with rare distributions were excluded. The similarity between station groups arising from cluster hierarchies was statistically evaluated using analysis of similarities (ANOSIM). A nonparametric permutational analysis of variance (PERMANOVA) on the Bray–Curtis similarity matrix (respiration rate data) was used to confirm differences in species structure between groups of clusters. To identify taxa responsible for differences between station clusters, SIMPER analysis was performed [92]. Furthermore, benthic communities were classified according to the dominant taxa (those contributing > 10% to the total respiration rate), with distinctive characteristic species forming the core of each community grouping. Cluster analysis based on the Jaccard index (using a presence/absence species matrix) was used to visualize differences in species composition between stations.

The structure of benthic communities was investigated by focusing on the life-history traits of prevailing taxa. Classification of life-history traits, such as feeding strategies, mobility, and reproductive behavior, was based on available literature [93,94,95]. A non-parametric permutational multivariate analysis of variance (PERMANOVA) was subsequently employed to assess differences in benthic abundance, biomass, and functional composition among communities.

To examine the relationships between environmental variables and patterns of benthic abundance, biomass, and diversity, redundancy analysis (RDA) was carried out. Preliminary detrended correspondence analysis (DCA) results indicated that the first axis length was less than three standard deviation units, suggesting that a linear approach such as RDA was more appropriate [96]. The environmental dataset included the aforementioned physical and sedimentological parameters, while five distinct biological datasets served as response variables. Two datasets represented the abundance and biomass of common species (those with FO > 50% and contributing ≥ 5% to total abundances). Two additional datasets included abundance and biomass for dominant functional groups (those exceeding 100 ind. m^−2^ in abundance or 1 g m^−2^ in biomass). The fifth dataset comprised diversity indices. To minimize the influence of extreme values, species abundance and biomass data were log-transformed. Forward selection procedures based on Monte Carlo permutation tests (with 999 permutations under the full model) were applied to identify environmental variables that most significantly influenced the observed biological patterns. Generalized linear models (GLM) with normal distribution and identity links were used to identify the effects of significant factors revealed by RDA on particular species. In addition, Pearson correlation coefficients were calculated to explore the relationships between selected environmental factors and benthic biological variables after testing the data for normality using the Shapiro–Wilk test. Statistical analyses were performed using PAST 4 and Canoco 4.5.

Mean biological and environmental values are presented with standard errors.

## 3. Results

### 3.1. Environmental Conditions

Stations associated with Transect I were situated relatively close to the coastline and were characterized by shallow depths, exhibiting a slight increase in depth from the inner to the outer regions of the fjord. These stations were significantly influenced by terrestrial runoff, with high concentrations of fine suspended particles. The local sediments predominantly consisted of fine-grained materials, with a substantial proportion of particles < 0.01 mm in size. A clear sedimentation gradient was not evident in this region, likely due to the relatively uniform distribution of glaciers and meltwater discharge along the coastline. The highest concentration of pelitic material and particles < 0.05 mm in size was observed in the central region of Storfjord (Table 1).

During the study period, near-bottom waters throughout the inner fjord were dominated by brine-enriched waters, characterized by low negative temperatures and high salinity (Table 1). An exception was observed at station 31, which exhibited transient hydrological properties.

Environmental conditions along Transect II were more heterogeneous, reflecting a stronger influence of Atlantic water, particularly in its southern segment. Stations along this transect were generally located in deeper waters, except for station 79, which was positioned near the sill at a shallower depth (Table 1). The greater distance from the shore reduced the influence of terrestrial inputs in this region, resulting in a lower proportion of fine-grained sediments (Table 1). Station 78(17), located in the inner fjord region, was influenced by winter water masses. Further out, station 79 was similarly affected, while stations 78(19) and 81 were subject to the influence of polar front waters. At station 80, a mixing of brine-enriched and polar front waters was observed, causing a slight increase in water temperature and a decrease in salinity. A distinct gradient in sea ice cover duration was apparent along both transects. In the uppermost fjord regions, up to station 31, IFP was six months, whereas stations beyond the sill experienced an ice-free period lasting nine months or longer (Table 1).

**Figure 2:**
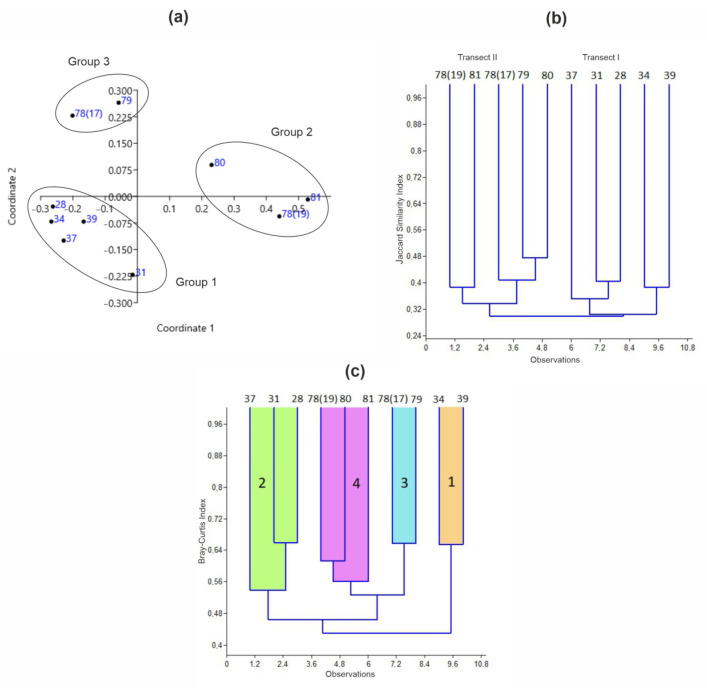
Non-metric multidimensional scaling plot of sampling stations in Storfjord in relation to environmental variables using Euclidean distance (**a**), dendrogram for cluster analysis based on the Jaccard index (**b**), and dendrogram for cluster analysis based on the Bray–Curtis similarity matrix calculated for benthic metabolic rate data (**c**). Different colors highlight different clusters.

nMDS analysis (Figure 2a) identified three distinct habitat types (ANOSIM: R = 0.971, *p* = 0.0004). Pelite content (28.2%) and water temperature (24.5%) were the primary contributors to the dissimilarity between Groups 1 and 2, whereas dissimilarity between Groups 1 and 3 was primarily driven by water temperature (40.2%) and pelite content (18.8%).

For Groups 2 and 3, temperature variations accounted for the majority of the dissimilarity (85.3%). Group 1 included all stations in Transect I, which were associated with winter water masses. This habitat type was characterized by relatively stable oceanographic conditions but was strongly influenced by the deposition of mineral-rich suspended sediment transported via meltwater. In contrast, the reduced degree of sedimentation along Transect II gave rise to two distinct habitat types: one influenced predominantly by polar front waters (Group 2) and the other shaped by brine-enriched waters (Group 3).

### 3.2. Benthic Diversity, Abundance, and Biomass

A total of 314 invertebrate taxa (Appendix A), distributed across 14 phyla, were identified at the 10 sampled stations. The Chao2 index estimated species richness for the study area to be 426 ± 29. The greatest diversity was observed among polychaetes (32% of the total number of taxa) and crustaceans (24%). Species richness was comparable between the two transects, with 232 species recorded in Transect I and 221 species in Transect II.

Cluster analysis clearly differentiated the benthic communities of Transects I and II (Figure 2b), showing a low degree of similarity (30%) between them. This separation was primarily influenced by distance from the shore, with depth having a relatively minor effect. Stations 78(17) and 79, located in the inner fjord closer to the shore, exhibited similar community compositions with the outer stations of Transect II. The most pronounced differences in taxa composition were observed between stations 34 and 39 and all other stations (Figure 2b). Despite these differences, measures of alpha diversity, species richness per sample, Shannon diversity (H′), Pielou’s evenness (J′), and the D_E_ index were similar across the sampled stations (Table 2).

The mean macrofaunal abundance within the study area was 6090 ± 560 ind. m^−2^, ranging from 3230 ind. m^−2^ at station 34 to 9680 ind. m^−2^ at station 81. There was an increase in abundance from the inner part to the outer part of the fjord (Table 2), although the difference between transects was not statistically significant (PERMANOVA, *p* = 0.11). The lowest abundances were recorded at stations 34 and 39 (Table 2). The mean biomass for the study area was 188 ± 17 g m^−2^, ranging from a minimum of 81 g m^−2^ at station 81 to a maximum of 466 g m^−2^ at station 37. Stations within Transect II exhibited higher similarity in terms of abundance and biomass compared to those in Transect I (Table 2), but the differences were not statistically significant (PERMANOVA, *p* ≥ 0.227). Benthic biomass showed a decreasing trend from the inner to the outer regions of Storfjord (Table 2), with values exceeding 260 g m^−2^ recorded closer to the fjord’s head. The mean biomass in Transect I was significantly higher than that observed in Transect II (PERMANOVA, *p* = 0.01). Average individual sizes of benthic organisms decreased progressively towards the seaward portions of the study area. This reduction in organism size was not associated with a shift toward r-strategist species, as evidenced by consistently negative D_E_ index values across stations (Table 2).

**Figure 3:**
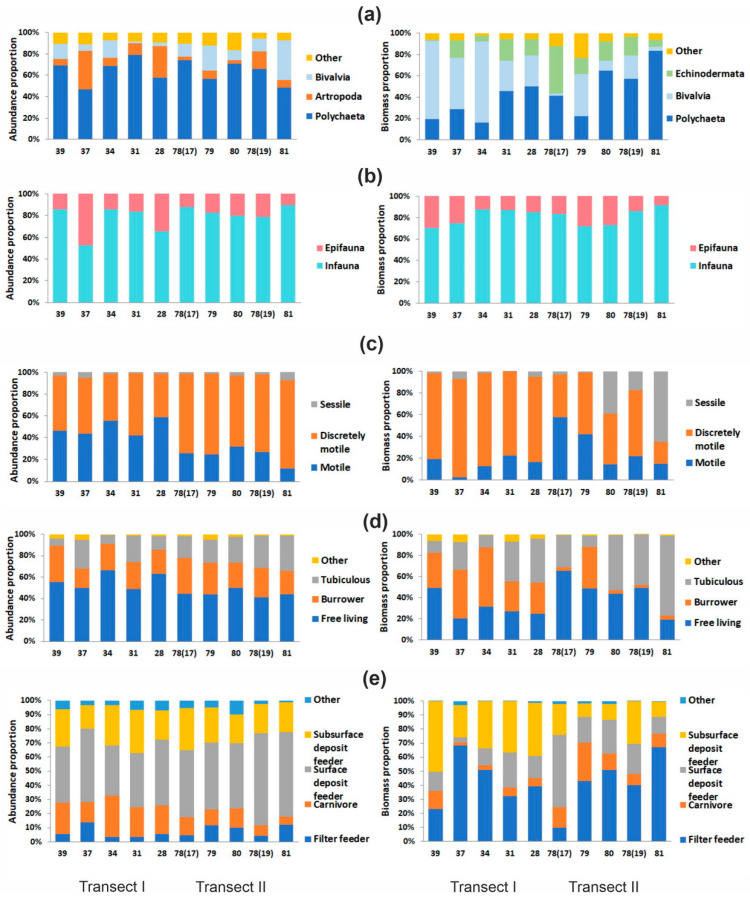
Structure of the benthos in Strofjord (main groups) according to benthic grab samples: taxonomic structure (**a**), position relative to the bottom surface (**b**), mobility (**c**), life habit (**d**), and trophic structure (**e**). Abundance (**left**) and biomass (**right**).

The abundance and biomass of major taxa exhibited significant differences between transects (*p* < 0.05). Polychaetes were the dominant group in terms of total abundance, contributing similarly to both transects (63–64%), although their spatial distribution patterns varied considerably, particularly in the inner regions of Storfjord (Figure 3a). Crustaceans were the second-most abundant taxa in Transect I (18%), whereas bivalves held this position in Transect II (19%). In terms of biomass, bivalves (50%) and polychaetes (32%) were predominant in Transect I, while Transect II was dominated by polychaetes (54%) and echinoderms (21%).

Significant differences between transects were also noted in the abundance and biomass of taxa characterized by varying mobility (PERMANOVA, *p* ≤ 0.007), the biomass of groups with different lifestyles (*p* = 0.03), and the biomass of trophic groups (*p* = 0.0084). Epifaunal taxa were more species-rich than infaunal taxa in both transects, accounting for 59% of species in Transect I and 53% in Transect II; however, infaunal organisms dominated in terms of both abundance and biomass (Figure 3b). Low-mobility species were dominant in species richness, comprising 43% of species in Transect I and 45% in Transect II. However, in areas close to the shore, mobile taxa showed greater dominance in abundance (49%), while discretely motile taxa were more prevalent along the fjord’s central axis (75%), forming a distinct and contrasting longitudinal gradient in dominance (Figure 3c).

In terms of biomass, discretely motile taxa were the dominant contributors, with their proportions being significantly higher at Transect I stations (75%) compared to Transect II (45%). An inverse relationship was observed between mobile and sedentary forms. Regarding lifestyle groups, free-living taxa were dominant in terms of species richness in both transects, comprising 37% in Transect I and 43% in Transect II, while also leading in abundance (Figure 3d). Their proportion was slightly higher in the shallow-water Transect I (57%) compared to the deeper Transect II (44%). In Transect I, free-living taxa and burrowing organisms equally contributed to the biomass (34% each), whereas in Transect II, free-living taxa (45%) and tubiculous animals (44%) were the largest biomass contributors.

The proportions of the key trophic groups in either abundance or biomass did not exceed 50–60% within the study area (Figure 3e). Surface deposit feeders had the highest abundance in both transects (42% in Transect I and 53% in Transect II), with their dominance being most pronounced at stations influenced by polar front waters (Figure 3e). In contrast, sub-surface deposit feeders demonstrated maximum abundance in the inner regions before the fjord’s sill. In terms of biomass, filter feeders were dominant, representing 42% of each transect. Notably, the contribution of sub-surface deposit feeders to total biomass was approximately twice as high in Transect I compared to Transect II, with peak values recorded at the fjord head. Conversely, the proportion of surface deposit feeders in biomass was higher in Transect II (Figure 3e). Carnivores showed a decreasing trend in abundance from the fjord head toward the open sea. A similar spatial decline was observed for the biomass of sub-surface deposit feeders, whereas surface deposit feeders exhibited increasing abundance toward the fjord’s mouth (Figure 3e). The spatial distribution of other trophic groups demonstrated more variable patterns, indicating complex community structuring across the sampled area.

The combination of ecological and behavioral traits identified 20 epifaunal and 23 infaunal functional groups. The largest number of species was recorded among free-living mobile epifaunal carnivores (48 species). Fourteen functional groups were represented by a single taxon, two groups by two taxa, and seven groups by three taxa. The total number of functional groups did not vary significantly between the transects studied (Table 2). Infaunal groups were the most abundant, with discretely motile burrowing surface deposit feeders (IDBsr; 20%) and free-living motile carnivores (IMFca, 12%) being particularly abundant in Transect I, while discretely motile tubiculous subsurface deposit feeders (IDTssd, 15%) dominated at stations in Transect II. The highest biomass was observed for IDTssd (19%) and discretely motile burrowing filter feeders (IDBsu, 17%) in Transect I, and for sessile tubiculous filter feeders (13%) in Transect II.

The zoogeographic structure of the benthic fauna was consistent between the two transects. Boreal–Arctic species comprised 78% of all species in Transect I and 79% in Transect II, with Arctic species almost twice as numerous (13% each) as boreal species (6% in Transect I and 5% in Transect II). Boreal–Arctic species also dominated in abundance, representing 64% and 66% of individuals in Transects I and II, respectively. Cosmopolitan taxa formed the second-most abundant group (19% in Transect I and 13% in Transect II). Boreal–Arctic species were also the primary contributors to biomass, accounting for 95% in Transect I and 90% in Transect II.

Approximately 31% of species were classified as rare, occurring at only a single sample. Six polychaete species were recorded across all samples (frequency of occurrence, FO: 100%) and, on average, dominated in abundance compared to other invertebrates (Table 3).

These species were *Lumbrineris mixochaeta* (9.5%), *Maldane sarsi* (7.6%), *Chaetozone setosa* (7.2%), *Leitoscoloplos acutus* (7.0%), *Galathowenia oculata* (6.9%), and *Heteromastus filiformis* (6.5%). Additionally, eleven species exhibited FO values ranging from 97% to 77%, while 15 other taxa were found with FO values between 70% and 50% (Table 3). Collectively, the species listed in Table 3 accounted for 81% of total abundance and 52% of benthic biomass.

The abundance of dominant species varied slightly between the two transects. In Transect I, *L. mixochaeta* (14.0%), *L. acutus* (9.0%), *M. sarsi* (8.4%), and *C. setosa* (7.1%) were most abundant, whereas in Transect II, *G. oculata* (12.1%), *H. filiformis* (10.1%), *Yoldiella nana* (8.3%), *M. sarsi* (6.8%), and *Mendicula ferruginosa* (6.6%) were the dominant taxa. Biomass was highest for the polychaetes *M. sarsi* (17.0%) and *Spiochaetopterus typicus* (12.0%) and the starfish *Ctenodiscus crispatus* (9.6%), as well as for the bivalves *Ciliatocardium ciliatum* (7.4%), *Tridonta borealis* (5.5%), *Yoldia hyperborea* (5.0%), and *Tridonta montagui* (4.0%). However, most of these species, except for *M. sarsi*, displayed relatively low FO values (less than 57%).

### 3.3. Benthic Communities

The ANOSIM test revealed the presence of four distinct clusters representing different benthic communities within the study area (R = 0.8919, *p* = 0.0003). Among these, Cluster 1 exhibited the most pronounced differences compared to the other clusters, while Clusters 3 and 4, both located in Transect II, showed the highest similarity (Table 4).

The cluster groups identified were significantly different from each other (PERMANOVA, *p* < 0.005 in all cases). In the inner basin, no clear spatial pattern was observed in the distribution of benthic communities. At the head of the fjord, a benthic community dominated by the bivalve *Yoldia hyperborea*—accounting for 17% of the total respiration rate—was identified in the winter water mass at depths of 89–97 m at two stations (Stations 39 and 34, Cluster 1 in Figure 2c). A comprehensive list of the other nine dominant species in this community is provided in Table 5.

This community was characterized by the lowest species diversity (118 species), low abundance (3700 ± 73 ind m^−2^), and relatively high biomass (227 ± 35 g m^−2^). The most abundant species were the polychaetes *Lumbrineris mixochaeta* and *Leitoscoloplos acutus*, which together made up 31% of the total abundance (Table 3 and Table 5). In terms of biomass, the bivalves *Y. hyperborea*, *Ciliatocardium ciliatum*, and *Bathyarca glacialis* were the major contributors, collectively accounting for 53% of the total biomass. From a functional perspective, this community was dominated by burrowing discretely motile surface deposit feeders (26%) and infaunal mobile free-living carnivores (22%) in terms of abundance. However, biomass was primarily contributed by discretely motile burrowing subsurface deposit feeders (24%) and discretely motile burrowing filter feeders (20%).

The polychaete *Maldane sarsi* was the dominant species in a significant segment of the inner and outer fjord regions affected by the brine-enriched water. Two distinct variants of this community were identified due to differences in environmental conditions. The first variant (Cluster 2 in Figure 2c), characterized by relatively high species richness (196 species), biomass (226 ± 30 g m^−2^), and abundance (6212 ± 707 ind m^−2^), was found at Stations 37, 31, and 28 in Transect I at depths of 83–115 m. In this community variant, *M. sarsi* accounted for 23% of the total community metabolism. The composition of dominant species differed considerably from that of the *Y. hyperborea* community (Table 5). The most abundant species in this community were *M. sarsi* and *L. mixochaeta*, each contributing 11% to the total abundance. Biomass was dominated by *M. sarsi* (30%), followed by the bivalves *Tridonta borealis* (14%) and *Astarte montagui* (13%). Functionally, the most abundant groups in this variant were discretely motile surface deposit feeders (19%), infaunal mobile free-living carnivores (16%), and motile free-living surface deposit feeders (16%). However, biomass was most strongly associated with discretely motile tubiculous subsurface deposit feeders, which contributed 31%.

Transect II exhibited a consistent gradient in benthic community composition. On the inner side of the sill, at depths of 130–157 m, another variant of the *Maldane sarsi* community was identified, with *M. sarsi* as the dominant species (15% relative respiration rate) and nemerteans as subdominants (14%) (Cluster 3 in Figure 2c). Consequently, the list of dominant species (Table 5) for this variant differed from that of the previous community. This community consisted of 143 invertebrate species, but featured lower biomass (159 ± 12 g m^−2^) and abundance (5700 ± 144 ind m^−2^) compared to the previous variant. The most abundant species were the polychaete *Heteromastus filiformis* (13%) and the bivalve *Yoldiella nana* (12%). In terms of biomass, the starfish *Ctenodiscus crispatus* (26%) was dominant, followed by *M. sarsi* (12%) and *Ciliatocardium ciliatum* (12%). From a functional perspective, abundance was primarily contributed by discretely motile burrowing surface deposit feeders (18%), discretely motile tubiculous surface deposit feeders (15%), discretely motile free-living surface deposit feeders (14%), and discretely motile tubiculous subsurface deposit feeders (14%). However, biomass was dominated by motile free-living surface deposit feeders (26%) and discretely motile burrowing filter feeders (18%).

The southern part of Transect II, located in the polar front zone at depths of 149–218 m, was inhabited by a community dominated by the polychaete *S. typicus*, which accounted for 29% of the total respiration rate in this area (Cluster 4 in Figure 2c). This community displayed moderate species richness (179 species), but its fauna consisted predominantly of small-sized animals, with the lowest recorded biomass (139 ± 18 g m^−2^) and the highest abundance (7820 ± 936 ind m^−2^). The most abundant species were the polychaete *G. oculata* (15%) and the bivalve *M. ferruginosa* (10%). Regarding biomass, *S. typicus* (40%) and *M. sarsi* (13%) were the dominant contributors. Functionally, the most important contributors to total abundance in this community were discretely motile tubiculous subsurface deposit feeders (23%) and discretely motile burrowing surface deposit feeders (17%). In contrast, biomass was dominated by sessile tubiculous filter feeders (40%).

### 3.4. Environmental Factors Shaping Benthic Communities

**Figure 4:**
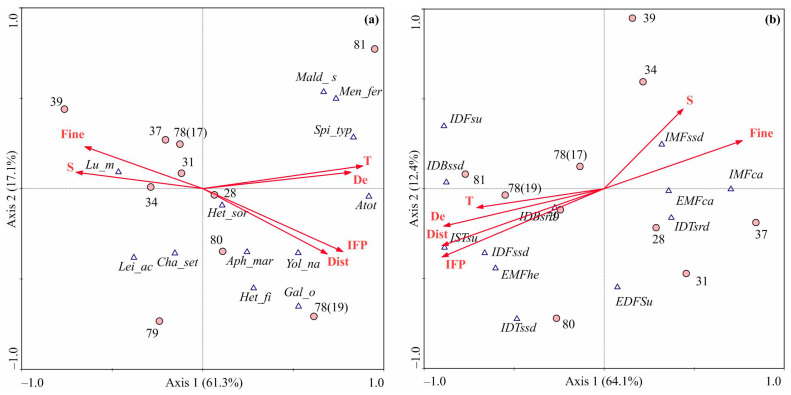
Ordination of stations by redundancy analysis with respect to abundance of the most common species (**a**) and abundance of the most common benthic functional groups (**b**) and their relations to environmental variables. Environmental variables: De—depth, T—water temperature, S—water salinity, Dist—distance to the nearest shore, Fine—the proportion of fine sediment < 0.05 mm, IFP—the length of the open water season. Biological variables: Atot—total benthic abundance, *Lu_m*—*Lumbrineris mixochaeta*, *Lei_ac—Leitoscoloplos acutus*, *Cha_set—Chaetozone setosa*, *Aph_mar—Aphelochaeta marioni*, *Het_fi*—*Heteromastus filiformis*, *Gal_o—Galathowenia oculata*, *Mald_s—Maldane sarsi*, *Spi_typ—Spiochaetopterus typicus*, *Men_fer—Mendicula ferruginosa*, *Yol_na—Yoldiella nana*, *Het_sor*—*Heterocyprideis sorbyana*, IDFsu—infaunal discretely motile free living filter-feeder, IDBssd—infaunal discretely motile burrowing subsurface deposit-feeder, IDBsrd—infaunal discretely motile burrowing surface deposit-feeder, IDTsrd—infaunal discretely motile tubiculous surface deposit-feeder, IDTssd—infaunal discretely motile tubiculous subsurface deposit-feeder, IDFssd—infaunal discretely motile free living subsurface deposit-feeder, IMFssd—infaunal motile free living subsurface deposit-feeder, ISTsu—infaunal sessile tubiculous filter-feeder, IMFca—infaunal motile free living carnivorous, EDFsu—epifaunal discretely motile free living filter-feeder, EMFca—epifaunal motile free living carnivorous, EMFhe—epifaunal motile free living herbivorous.

The RDA model, conducted using abundance data, yielded statistically significant results, as verified by the significance test of all canonical axes (F-ratio = 3.73, *p* = 0.012). The first two axes collectively accounted for 79.4% of the total variance, with Axis 1 explaining 62.3% and Axis 2 contributing 17.1%. Axis 1 exhibited a positive correlation with IFP, distance from shore, and depth, successfully distinguishing between stations with higher abundance (on the right side) and those with lower abundance (on the left side) (Figure 4a).

The Monte Carlo test indicated that IFP was the only factor significantly explaining the observed patterns (Table 6), with positive effects on total abundance and on the abundance estimates of several common species, including *H. filiformis*, *G. oculata*, *S. typicus*, *Y. nana*, and *M. ferruginosa* (GLM, *p* < 0.05), which collectively contributed 25% to the total abundance. Conversely, IFP negatively affected the abundance of the carnivorous polychaete *L. mixochaeta* (GLM, *p* < 0.05). Other common species, such as *Aphelochaeta marioni*, *C. setosa*, *L. acutus*, *M. sarsi*, and *Heterocyprideis sorbyana*, accounting for 33% of the total abundance, were not associated with a specific habitat, a finding further corroborated by correlation analysis.

The RDA model based on the abundance of functional groups explained 91.2% of the total variation (F-ratio = 5.18, *p* = 0.013). Axis 1 accounted for 64.1% of this variance, separating stations into groups dominated by carnivorous species on the right and by infaunal sedentary tubiculous filter feeders and discretely motile free-living subsurface deposit feeders on the left (Figure 4b). This first axis was negatively correlated with all factors except salinity and the presence of fine sediment fractions. The Monte Carlo test revealed that two factors—IFP and distance from shore—significantly explained the total variation (Table 6). Generalized linear models indicated a significant positive effect of IFP on the abundance of burrowing, free-living, and tubiculous subsurface deposit feeders (IDBssd, IDFssd, IDTssd), as well as on sedentary tubiculous filter feeders (ISTsu) (*p* < 0.05). However, IFP had a negative effect on discretely motile free-living infaunal carnivores (IMFca) (GLM, *p* < 0.05).

RDA models constructed using biomass data were not statistically significant, with the first axes explaining less than 40% of the total variation for each model. Nonetheless, correlation analysis revealed a negative impact of factors associated with trophic conditions (*p* < 0.02) and a positive influence of fine sediment proportions (*p* = 0.04) on total biomass. Moreover, the biomass of sedentary tubiculous infaunal filter feeders increased in the outer part of Storfjord, positively correlating with depth, temperature, distance from shore, and IFP (*p* ≤ 0.01), while exhibiting a negative correlation with fine sediments (*p* = 0.008). The biomass of free-living discretely motile subsurface deposit feeders increased with distance from shore and increasing IFP (*p* ≤ 0.03), whereas the biomass of discretely motile free-living epifaunal filter feeders was negatively correlated with water temperature (*p* = 0.037).

The RDA model based on diversity indices was found to be insignificant (F-ratio = 0.64, *p* = 0.725).

## 4. Discussion

### 4.1. Environmental and Trophic Conditions in Storfjord

The inner region of the fjord, which is isolated by the sill and influenced by Arctic-origin waters, is characterized by a relatively stable hydrological regime in its bottom layer. To date, there have been no documented instances of significant intrusion of Atlantic-origin waters into the interior of the Storfjord beyond the sill [98]. This hydrological stability distinguishes Storfjord from western fjords, where the penetration of warm water into the innermost basins has been observed [23,99]. The sill plays a critical role in maintaining cold, dense, and oxygen-rich bottom water, a condition largely attributed to rapid ice formation [74,81,100,101]. The inner section of the fjord, situated in the front of the sill, acts as a reservoir for dense brine-enriched water, effectively preventing its outflow due to the influence of cyclonic circulation. This leads to the formation of a dome-like structure that extends upward to a depth of 90 m [98].

In contrast, the hydrological conditions on the seaward side of the sill exhibit greater temporal variability. The polar front, located over the outer section of the fjord, lacks a fixed position and undergoes seasonal shifts either northward or southward [76]. These dynamic shifts result in rapid water mass transformations. The influence of Atlantic-origin waters on the outer Storfjord is weaker compared to the west coast of Svalbard, where a more pronounced temperature gradient along the fjord axis is observed. In western fjords, summer bottom temperatures range from 1.9 to 3.5 °C [19,42,65,99,102,103], occasionally reaching as high as 5 °C during episodic Atlantic water inflows [99]. By contrast, the annual variability in near-bottom temperatures in the outer Storfjord is less pronounced than in the western fjords of Svalbard but greater than in the inner Storfjord.

The hydrological conditions in the outer section of the study area are governed by two primary factors: the unstable position of the polar front and periodic overflow of brine-enriched waters from the inner Storfjord. These brine overflows across the sill typically occur during ice production, when new brine water is formed, or under the influence of strong wind events [74,104]. This phenomenon begins between January and mid-March, with a cessation between mid-August and late September [104]. The duration of the overflow is typically approximately 180 d, though its occurrence can be intermittent. In July 2017, cold, saline waters of Arctic origin were recorded as far as station 80, while the influence of warmer waters associated with the polar front was observed at the outermost two stations along Transect II. However, as previously discussed, the properties of the bottom layer at these stations exhibit significant interseasonal and interannual variability. Generally, hydrological conditions beyond the sill are subject to relatively abrupt variations, with rapid transitions from weakly positive to strongly negative temperatures.

The bottom sediments along the transects in Storfjord are generally homogenous in composition, predominantly consisting of mud. However, slight variations in the ratios of pelitic and aleuritic fractions within the mud are evident across the sections. Notably, a higher percentage of fine sediment fractions is observed at stations situated near the shoreline along Transect I. This proportion decreases progressively with increasing distance from the shoreline, reflecting a general fining-to-coarsening trend. These findings are consistent with those of Fossile et al. [84], who reported an increase in grain size and sand fraction from the inner to the outer regions of Storfjord in 2016.

The sediment characteristics and stability of sediment deposition in Svalbard fjords are influenced by the sedimentation rate, which itself is governed by factors such as meltwater input and proximity to shore. Periods of elevated temperatures have been associated with increased terrestrial and glacial runoff, which in turn intensifies water turbidity, the input of terrestrial bioavailable carbon, and rates of carbon burial [82,105]. In the Storfjord region, turbidity plumes frequently extend far from the shore [106], creating a distinct gradient of turbidity that transitions from the shoreline to the fjord’s central zone, and from its inner to outer regions. Stations located nearshore exhibited higher mineral suspension loads relative to those situated farther offshore, as evidenced by the elevated pelite-to-aleurite ratio observed in their sediments. Current estimates of sedimentation rates in the northern and central sections of the fjord are approximately 3.6 mm yr^−1^ [84], a rate lower than that observed in western fjords (25 mm yr^−1^), particularly glacier-fed systems [107], yet higher compared to rates reported for the Storfjord area in 2001 (1.8 mm yr^−1^) [87]. This increase in sedimentation rate is attributed to enhanced terrestrial runoff and sediment loads associated with heightened glacial melt and increased freshwater discharge [82,105]. Furthermore, this phenomenon has been amplified by rising air temperatures over the Svalbard Archipelago, a trend that has persisted since the mid-1990s [21,108], resulting in intensified and prolonged glacier melt.

In western Svalbard fjords, increased inflow of Atlantic-origin waters has led to warming of the water column, enhanced salinity, and a reduction in seasonal sea ice cover, with complete ice-free conditions observed during some years [23,105,109]. In contrast, Storfjord has demonstrated relative stability in its oceanographic and cryospheric conditions over recent decades. The fjord maintains persistent seasonal sea ice cover and continues to play a crucial role in the production of cold, dense, and saline bottom waters, a significant contributor to Arctic Ocean ventilation. Despite this general stability, the increased inflow of Atlantic Water into Storfjord, combined with rising air temperatures over the archipelago, has resulted in a modest reduction in the duration of seasonal ice cover, as indicated by comparative analyses of historical data spanning 1970 to 1990 [110]. In the 2010s and 2020s, the onset of ice formation in Storfjord has shifted 2–3 months later, typically occurring in January or February, as opposed to the cooling phase of the 1970s and 1980s, when freeze-up began as early as November or December. Similarly, the ice-free season has begun earlier, starting in June rather than July or August, and the duration of the ice-free period in the outer fjord has extended to as much as nine months. This prolonged open-water period has increased the influence of atmospheric processes on the water dynamics of the region. Enhanced wind-driven mixing and disruptions to surface stratification have become more prominent during the ice-free season, promoting vertical mixing of the upper water column [111]. Additionally, the extended open-water period has contributed to wind forcing, resulting in more pronounced instances of brine overflow events [104].

An increase in the duration of the open-water season in ice-covered seas has been linked to enhanced primary productivity of pelagic algae [112,113,114,115]. In Svalbard fjords, the seasonal cycle of phytoplankton blooms follows the melting of sea ice and the termination of ice algal blooms, with production peaking during the nearshore phytoplankton bloom and subsiding with the onset of the polar night in October [49]. An earlier onset of ice melt has the potential to benefit benthic ecosystems by reducing the duration of periods with low food availability. This is achieved by extending the window of pelagic primary production and coinciding with the earlier influx of terrigenous detritus. However, the delivery of primary productivity to the seafloor is influenced by a range of controlling factors, including hydrodynamic and biological processes.

Increased meltwater input into fjords contributes to greater turbidity, which results in a reduction in the depth of the euphotic zone. Additionally, it intensifies flocculation and sedimentation processes that reduce the proportion of organic matter reaching the seafloor [105]. Fjords with tidal glaciers or high riverine inflows often display strong spatial gradients in primary production, with lower phytoplankton productivity near ice or river sources and higher productivity in the outer fjord due to turbidity gradients [49,116,117]. Zooplankton activity also exerts a notable influence on the downward flux of organic matter, as grazing can substantially reduce the delivery of fresh organic inputs to the benthos. For instance, in some regions along the east coast of Greenland, increases in phytoplankton production have led to reduced planktonic detritus reaching the seafloor due to heightened zooplankton grazing [118]. In Storfjord, the earlier onset of phytoplankton blooms, driven by earlier ice melt, an influx of terrestrial nutrients, and increased nutrient advection from depth, contributes to elevated primary production across the fjord. This productivity generally follows a gradient, increasing from the apex towards the outer regions and from the shores towards the central parts of the fjord. In 2016, a chlorophyll *a* concentration gradient was observed in the bottom sediments, with concentrations peaking in the middle and outer parts of the fjord at depths less than 200 m, while deeper waters, exceeding 300 m, exhibited lower concentrations. This pattern was attributed to the reduced delivery of fresh organic material originating from microalgae to the seafloor at greater depths [84].

Organic matter reserves in the sediments of Storfjord are critical for the Svalbard coastal ecosystem, supporting benthic productivity and acting as a potential carbon sequestration mechanism. Higher organic carbon content (6.8–7.7 mg C g^−1^) has been recorded in sediments in the middle and outer parts of the fjord, while lower concentrations (5.5 mg C g^−1^) are evident in the fjord’s apex. This disparity is attributed to the dilution of organic matter by mineral particles, which is particularly pronounced under high turbidity conditions [84]. The quality of organic matter in Storfjord exhibits spatial variability. Summer melting of tidal glaciers drives significant inflows of terrigenous material to the apex of the fjord, with up to 70% of the organic matter there being of terrestrial origin. This proportion gradually decreases to 59–53% in the middle and outer parts [87]. The increased turbidity associated with nearshore terrestrial inflows restricts primary production, contributing to the dominance of refractory organic matter in these areas. In terms of transect-specific characteristics, sediments along Transect I, being nearer to the shoreline, are strongly influenced by terrigenous inputs resulting from glacial melt, making terrestrial organic matter the predominant contributor. On the other hand, Transect II, located farther from the coast, is less affected by turbidity and benefits from greater penetration of sunlight and availability of nutrients, fostering increased input of fresh marine detritus.

### 4.2. Current State of Soft-Bottom Benthic Communities

Our research on the benthic environments of Storfjord revealed a considerable diversity of soft-bottom fauna at depths exceeding 80 m. The communities were dominated by boreal–Arctic species but also included a notable presence of Arctic and, to a lesser extent, boreal species. The benthic fauna showed a high functional diversity, with abundance and distribution patterns varying according to habitat conditions. Despite the fluctuating environmental conditions across the fjord, no distinct signs of stress were observed in the benthic communities. The trophic structure was relatively balanced, displaying only weak dominance among the main trophic groups—an observation that appears to align with patterns typical of coastal systems. The benthic fauna composition was dominated by infauna rather than epifauna, suggesting that water column dynamics, such as strong currents, were relatively subdued in the studied areas [119].

The dominance of polychaetes and arthropods in the soft-bottom communities is a well-documented characteristic of deepwater habitats at high Arctic latitudes [28,51,120,121]. This pattern has also been widely observed in the mouth regions of fjords across the Svalbard archipelago [42,54]. Interestingly, the benthic fauna of Storfjord appears to exhibit greater biodiversity compared to smaller fjords in western Spitsbergen. Using the Chao2 diversity index, our study predicts the presence of over 100 additional invertebrate species not yet identified in the available records. Alpha diversity in Storfjord was approximately 40% higher than that observed in other western fjords, such as Hornsund Fjord, Grønfjord, Isfjord, Sassenfjord, Noor-Fjord, and Bellsund Bay, where species richness generally ranges from 55 to 70 species per 0.3 m^2^, with an average of 60 species per 0.3 m^2^ [42].

A clear pattern in biodiversity was observed with increasing depth, from 83 to 218 m. Nearshore habitats differed significantly in species composition compared to deeper water transects. In the inland basin, such as Station 78 (isolated by the sill) species composition showed similarities to other Transect II stations. However, in general, inshore benthic communities displayed notable differences in both species richness and community composition compared to shelf communities [122]. These differences appear to be driven by the physical isolation of certain fjordic zones, the varying contributions of organic and inorganic sediments, and the unique trophic dynamics within each habitat. The relatively high faunal diversity observed in our study area, which features a monotonous topography, could be attributed to the organic enrichment of benthic sediments and the relatively low levels of disturbance from inorganic sediment deposition compared to smaller fjords.

Most of the species listed in the top ten most abundant taxa in Table 3 are widespread opportunistic species recognized for their prevalence and ecological importance in soft sediment environments. These species have been extensively documented as dominant and often key contributors to benthic communities not only in the fjords of Svalbard [39,42,49,50,54,57,64,123,124,125] but also in fjords and coastal regions of Norway and the Kola Peninsula [121,126,127,128]. The widespread distribution of these species can be attributed to their high tolerance to variations in water temperature and their reproductive strategy, which involves short-lived lecithotrophic larvae. Such adaptation allows them to thrive in cold, seasonally unfavorable environments with prolonged periods of low availability of fresh food [125,129]. This life strategy is a critical ecological trait, enabling survival and even dominance in Arctic fjord ecosystems where seasonal and environmental extremes are the norm [130].

Interestingly, an opposing trend in the abundance of benthic fauna was observed along the axis of Storfjord compared to the spatial patterns documented in the glacial fjords of western Spitsbergen. Generally, in glacial fjords, benthic abundance tends to increase from the outer to inner regions, except near the glacier fronts where diversity and abundance decline due to harsh living conditions such as high sedimentation and limited habitat suitability [41,42,54,131]. In these western fjords, biomass peaks in estuarine areas and declines in the inner basins, a pattern likely shaped by gradients of freshwater input, sediment load, and nutrient delivery, which drive complex habitat heterogeneity near glaciers [41,42]. In contrast, the environmental dynamics in Storfjord present a different scenario. Here, the axes of spatial variability reflect variations in sedimentation, hydrological stability, and trophic dynamics, with a decrease in biomass and an increase in abundance observed moving seaward along the central axis of the fjord.

Elevated biomass values in Storfjord were identified in the upper regions of the study area, particularly in areas dominated by large-sized bivalves such as *Y. hyperborea*, *E. tenuis*, and *C. ciliatum*. These bivalves contribute significantly to the overall biomass due to their size and longevity, traits that enhance their ability to capitalize on organic materials settling into the benthic zone. As one moves seaward along the fjord’s axis, biomass decreases while abundance increases, indicative of a shift in the community structure. This transition aligns with the conditions in the outer areas of the fjord where fluctuations in hydrological parameters, especially in the polar front zone, create a less stable environment. Here, rapid changes in temperature, salinity, and nutrient availability likely favor smaller opportunistic species, which are adapted to capitalize on such dynamic conditions. The distribution of dominant taxa in the central areas of Storfjord aligns with patterns previously described for western Svalbard fjords, where polychaetes dominate in terms of abundance while both polychaetes and bivalves dominate in terms of biomass [42].

At the stations studied, a relatively homogenous distribution of benthic abundance and biomass was observed at depths exceeding 80 m. This homogeneity likely reflects the stable topographical features of the seabed at these depths, coupled with the reduced influence of negative factors such as trawling and predation. It is worth noting that trawling during the ice-free season exerts only a minor impact on the southern segment of the outer Storfjord [132,133,134]. The commercial stocks of demersal fish species in the Svalbard region are concentrated primarily around west Svalbard waters. In contrast, the deep waters of Storfjord are predominantly inhabited by Arctic polar cod (*Boreogadus saida*), a planktivorous species that exhibits a limited benthic dependence for feeding [135]. Marine mammals in the Storfjord area, walruses and sea hares, rely on benthic prey but primarily feed at shallower depths, leaving the deeper zones less impacted by top-down ecological pressures [136].

The functional structure of the benthos in the area displayed a gradient that correlated with the siltation gain gradient. Specifically, as the content of sediment instability decreased, a parallel reduction in the abundance of mobile, free-living taxa were observed, alongside a rise in sedentary tubiculous species. This trend likely reflects the favorable habitat conditions for tube-dwelling and burrowing species while limiting the mobility of free-living fauna. The diversity in functional groups within the examined depth range along transects indicates that the ecological niches available within the study area are being effectively utilized.

### 4.3. Distribution Patterns of Benthic Communities

In western Svalbard fjords, certain patterns in benthic infaunal communities are well-documented and strongly associated with the characteristics of the prevailing water masses [40]. These communities are often structured by gradients in sedimentation and nutrient conditions, reflecting the influence of dynamic pelagic-benthic coupling and terrestrial inputs [41]. A comparable scenario was observed in the deeper waters of Storfjord, where community composition and distribution were closely associated with variations in water mass properties, sediment deposition, and their interactions with pelagic processes. These dynamics contribute to distinct spatial patterns, illustrating how fjord ecosystems integrate and respond to environmental gradients across benthic and pelagic domains.

Among the benthic species identified in Storfjord, the bivalve mollusk *Y. hyperborea* stands out due to its wide distribution in the northern hemisphere and its preference for environments with mud sediments. This species thrives in shallow-water and mid-depth zones and derives its nutrition primarily from sediment surface detritus, although it is capable of supplementing its diet with suspended particles during periods of heightened microalgal deposition [137]. Regions influenced by terrestrial or glacial runoff often host large populations of *Y. hyperborea*, which is well-adapted to elevated sedimentation rates and suboptimal feeding conditions [54,137,138,139]. While the prevalence of *Y. hyperborea* is sporadically documented in other fjords of western Svalbard [40], its distribution exhibits significant variations based on temperature and sediment patterns. Interestingly, despite typically avoiding waters with subzero temperatures [140], *Y. hyperborea* was recorded in Storfjord at depths up to 100 m in brine-enriched waters—a finding that shows its potential adaptability to Arctic conditions.

The *Y. hyperborea*-dominated benthic community was primarily composed of non-carnivorous taxa, including *L. acutus*, *E. tenuis*, and *M. sarsi*. These taxa function primarily as sub-surface deposit feeders, heavily reliant on organic carbon stores within the sediment for sustenance. In comparison to other benthic communities found within Storfjord, the *Y. hyperborea* community demonstrated the lowest proportional contribution of microalgal-derived carbon to its total organic matter budget. This suggests a significant reliance on terrigenous detrital inputs, characteristic of stable, sediment-enriched environments where primary production plays a more minor role in the sustenance of deeper benthic fauna.

During the 1920s, a period characterized by significant cooling and reduced terrestrial and glacial runoff, the bivalve *Y. hyperborea* was recorded in dredge samples from the inner Storfjord. However, its abundance at that time was not noteworthy, and it failed to dominate the biomass in this region [70]. This observation suggests that *Y. hyperborea* tends to achieve higher abundance and biomass during warmer climatic conditions. The increased sedimentation rate in this area appears to adversely affect the abundance of the tubiculous polychaete *M. sarsi*, a competitor of *Y. hyperborea* for both space and food resources [139]. Since the composition and abundance of benthic fauna as well as community structure are generally recognized as direct indicators of environmental gradients [119], we assume that stronger sedimentation was typical only for stations 34 and 39 among all nearshore locations, despite sediment grain size distribution data failing to confirm this observation [85]. The reduced species richness and low abundance of benthic fauna at these stations indicate a relatively higher turbidity, impacting community dynamics. Specifically, the abundance of *M. sarsi* was found to be five times lower at these stations compared to other locations within Transect I at similar depths. This species is known to be sensitive to increased sedimentation; it tends to disappear from the inner regions of the western fjords of Svalbard [125].

The distribution of *M. sarsi*-dominated communities is typically concentrated in areas subject to moderate to weak sediment loads, particularly in regions influenced by winter water characteristics. This polychaete is broadly distributed across the World Ocean, frequently reaching high abundances in habitats characterized by soft muddy or muddy-sandy substrates. In the Svalbard coastal zone, *M. sarsi* populations are prevalent in the cold waters of inner fjords and bays that feature stable sediments with moderate to low sediment loads [41,42]. Recognized as a key bioturbator, *M. sarsi* plays an important role in the ecosystem; its feeding activities enhance the transfer of buried organic carbon from deeper sediment layers to the surface, promoting oxygenation of the sediments beneath and thereby fostering increased benthic diversity [41,141]. In communities where *M. sarsi* is dominant, elevated abundances have been recorded not only among subsurface deposit feeders, such as *M. sarsi* and *H. filiformis*, but also among surface deposit feeders including *C. setosa*, *A. marioni*, *P. arcticus*, and *Y. nana*. This distribution pattern reflects increased inflows of organic matter from the pelagic environment, which supports diverse benthic assemblages. During the 1920s, *M. sarsi* was one of the prevalent species in the inner Storfjord, particularly abundant in the southern portion of the inner basin, where it exhibited substantial biomass at depths ranging from 80 to 160 m [70].

A community dominated by the polychaete worm *S. typicus* was identified in the shelf region of Storfjord, specifically within the polar front zone. *S. typicus* is widely distributed throughout the Arctic and in Svalbard waters, where it reaches high abundance in fjord mouths and along the open coast, particularly in areas influenced by Atlantic waters that are characterized by low sediment loads, as well as in conditions associated with an increased supply of marine organic matter [41,42]. Within the middle section of the Barents Sea shelf, this species is consistently identified as one of the most prevalent benthic species [142,143,144,145]. *S. typicus*, whose tubes extend several centimeters above the sediment surface, is believed to primarily feed on suspended sediment. However, at greater depths, it may adapt its feeding strategy to include the collection of detritus [94]. The feeding strategies of other abundant species within this community are diverse, targeting fresh detritus of microalgal origin from various sources, including the sediment surface (*G. oculata*, *C. setosa*, *A. marioni*, *Y. nana*), the sediment column (*M. sarsi*, *H. filiformis*), or through suspension feeding *(M. ferruginosa*). This diversity confirms the critical role of marine phytoplankton as a substantial source of organic matter. At the same time, the abundance of *S. typicus* was comparatively low, being nearly four times less than that of *M. sarsi*. While *M. sarsi* exhibited densities similar to those of the initial community observed in the inner fjord, current conditions of environmental instability are reflected in the presence of younger, smaller specimens.

The distribution patterns of both variants of the *M. sarsi* benthic community highlight the stability of environmental conditions prevailing in most of the inner Storfjord during periods of both Arctic warming and cooling. The functional structure of this community indicates the significant role of buried terrigenous organic matter as a nutritional resource for benthic fauna. The *S. typicus* community at the mouth of the fjord, located within polar front waters, serves as a transitional form bridging coastal and shelf marine ecosystems. In terms of species composition, it shows considerable similarity to the deeper shelf bottom fauna of the Barents Sea, while its functional structure and numerical dominants reflect a mixed composition of food resources. In contrast, the *Y. hyperborea* community, found at depths of 80–100 m at the top of the fjord, appears to be of a more transient nature. This community has been observed to exist outside of the shallow water zone during warmer climatic periods in the Arctic. Intense coastal runoff has been recognized as a crucial factor in the formation of this community, effectively sorting bottom invertebrates based on their resilience to increased sedimentation. A relationship between benthic community composition and distribution and high inorganic suspended sediment loads has been observed in other Arctic localities, such as the Canadian coastal zone and the Novaya Zemlya archipelago [38,46]. This sorting phenomenon has been linked to a notable decline in species richness and abundance within the benthic fauna, further illustrating the complex interactions between environmental conditions and community dynamics in the Storfjord ecosystem.

### 4.4. Environmental Drivers of Macrozoobenthos

The infaunal benthic communities of Storfjord are primarily regulated by the vertical carbon flux from the euphotic zone and terrestrial runoff. The interaction between these closely linked factors determines the availability of fresh organic matter for deposit feeders—a critical resource for growth, reproduction, and survival, particularly for juvenile benthic organisms during periods of seasonal food scarcity [146,147]. Organic matter of terrestrial origin plays a particularly significant role in supporting deposit feeders in nearshore zones.

In Arctic and subarctic fjords, glacial activity and coastal runoff shape the distribution of benthic organisms [41,53]. In Svalbard fjords, species richness and abundance decline near glacier fronts [41,53]. High suspended mineral particles bury benthic larvae, clogging their tubes and filters, and diluting the sediment organic content [148,149,150]. Elevated mineral suspension destabilizes sediments, impeding organisms’ ability to burrow and maintain body/tube stability [54,150]. Although less extreme than near glacier fronts, coastal Transect I still experiences high sedimentation, resulting in a decline of sedentary species and an increase in mobile species. At 34 km offshore along Transect II, there is a significant change in species composition: the proportion of mobile species decreases by 50% and sedentary species nearly double in abundance, while tubiculous organisms increase.

Sedimentation also exerts a significant influence on the feeding conditions of benthic fauna. The low temperatures in the inner Storfjord contribute to reduced microbial mineralization of organic matter, allowing for the rapid burial and subsequent preservation of organic material. This accumulation of organic matter in bottom sediments functions as a “food bank”, providing a stable reservoir of labile organic carbon that persists despite seasonal or interannual variability in vertical detrital flux [151]. The stability of this organic carbon reservoir enhances the resilience of benthic communities, particularly detritivorous taxa, to seasonal fluctuations in primary production. This resilience supports benthic activity during the unproductive polar night [48,152,153,154,155].

Environmental parameters such as temperature, salinity, depth, and the proportion of fine sediments showed no significant effects on the benthic communities in Storfjord (Table 6). The predominance of eurythermal species capable of tolerating fluctuations in salinity likely explains the minimal influence of thermohaline characteristics. This observation contrasts with patterns in western Svalbard fjords, such as Hornsundfjord, where near-bottom temperatures have been shown to significantly impact the distribution of benthic fauna [103], or fjords further north, where water temperature strongly affects megabenthic epifaunal communities [43]. In most glacier-influenced fjords of Svalbard and Novaya Zemlya, sedimentation dynamics and food availability are also regarded as more critical determinants of benthic community structure than water temperature [46,54].

Bottom topography is widely recognized as a critical factor in shaping benthic community structure [156]. However, in the context of Storfjord, where the seafloor is characterized by relatively uniform muddy sublittoral sediments, the particle size distribution has a negligible impact on local benthic fauna. This pattern extends to other Arctic fjords with similar sedimentary environments, where the stability and coherence of muddy substrates are important in supporting benthic ecosystems [46,54].

Although depth is not an ecological factor per se, it plays an important role in determining various habitat characteristics. Along the Svalbard coast, for example, foraging conditions, which are generally negatively correlated with depth and influenced by water mass characteristics, often exhibit pronounced vertical patterns [43]. Research shows that organic matter (such as phytoplankton or zooplankton fecal pellets) breaks down during descent, with deeper depths and longer submergences speeding up this process [157,158]. In Storfjord, there were about five times less phytoplankton pigments exported to the benthic layer at >300 m, showing depth-dependent degradation and transformation. However, the decline in total organic matter was less at deeper depths compared to shallower stations [84].

Oxygen availability, an important driver of benthic organism distribution in many environments, was not explicitly analyzed in this study. It has been established that ice-covered fjords exhibit sufficient oxygenation, which is attributed to seasonal vertical mixing processes [111]. Previous research has demonstrated that the lower layers of Storfjord are adequately ventilated [84].

## 5. Conclusions

Storfjord is characterized by a weak oceanographic gradient along its central axis, influenced by the expansive presence of cold, brine-enriched water and limited intrusion of Atlantic water. The inner, cold-water part of the fjord exhibits relatively stable water conditions, whereas the outer section beyond the sill is marked by more variable and stressful environmental conditions for benthic populations. The fauna of Storfjord, as studied along its central axis, demonstrates significant functional diversity, efficiently utilizing the ecological niches available on the soft seabed. Notably, the benthic diversity in Storfjord is higher than that of the western fjords of Svalbard, attributed to the higher input of organic carbon. The benthic communities in Storfjord are dominated by polychaetes, crustaceans, and bivalves in terms of abundance, while biomass is largely contributed by polychaetes, bivalves, and echinoderms. The most abundant species in this fjord are highly tolerant of low water temperatures and well-adapted to enduring extended periods of fresh food scarcity during the polar night. The spatial distribution of benthos is closely linked to environmental factors such as the volume of coastal runoff (distance to shore) and the duration of the ice-free period. Along the fjord’s central axis, an increasing abundance gradient and a decreasing biomass gradient are observed as one moves toward the open sea. Biological factors, such as predation, play a minimal role in structuring benthic communities in Storfjord, largely due to the low abundance of benthivorous taxa. Instead, benthic communities are shaped by physical and environmental factors, including water mass properties, the strength of benthic–pelagic coupling, and sediment load. The stable hydrological parameters, combined with seasonal variations in moderate to negligible inputs of mineral suspension and terrigenous organic matter support relatively high benthic biomass. Key species within the study area include the polychaete *Maldane sarsi*, which exhibits moderate sensitivity to environmental variations, and the bivalve *Yoldiella hyperborea*, which demonstrates a lower sensitivity to such variability in the inner Storfjord. In contrast, the polychaete *Spiochaetopterus typicus* is prevalent in the outer region of the fjord, thriving under conditions characterized by weak sedimentation and a heightened contribution of pelagic plankton to the total organic matter. These structural characteristics of the Storfjord benthic communities provide indirect but time-integrated insights into the quantity and quality of organic material reaching the seafloor, as well as variations in sedimentation and organic burial rates. The species composition and community structure of *Y. hyperborea* differ most significantly from other benthic communities in Storfjord. The presence of abundant populations of this shallow-water, sediment-resistant species at depths exceeding 80 m is considered a temporary phenomenon, likely triggered by the intensification of glacial melting over the past 20 years. Consequently, this mollusk can serve as an indicator species, reflecting the intensity of glacial runoff, which varies across different climatic phases. Our data contribute to the current understanding of the functioning of modern cold-water benthic fauna in Svalbard and establish a foundation for long-term monitoring of Arctic seabed ecosystems under conditions of climate change. The benthic communities of Storfjord offer valuable insights into ecological dynamics associated with increased or decreased glacial melting, as well as changes in the duration of ice-free periods. This information can be instrumental in modeling ecological responses to future environmental shifts in Arctic marine systems. The area of the Svalbard coast selected for research may serve as a model; however, to comprehensively understand the biological processes occurring in polar regions under the influence of climate change, it is essential to expand the geography of seafloor community research in both the Arctic and Antarctic.

## Figures and Tables

**Table 1:** Environmental characteristics of sampling stations in Storfjord. Stations are arranged in a north–south geographical orientation.

St.	CC	L	D	T	S	WM	IFP	Sediment	AC	PC	SC	LOI
Transect I												
39	78°22.04′ N 19°38.78′ E	14	89	−1.71	35.28	BSW	6.2 ± 0.4	Light brown mud, black and gray clay, gravel, pebbles	52.8	40.1	88.7	9.4
37	78°11.71′ N 19°24.57′ E	9	83	−1.73	35.32	BSW	6.4 ± 0.4	Light brown mud, gray clay, sand in the lower layers, pebbles	40.3	43.1	81.0	8.2
34	77°59.97′ N 19°12.00′ E	10	97	−1.79	35.34	BSW	6.6 ± 0.5	Light brown mud, black and gray plastic clay, a small pebbles	54.8	37.4	88.6	9.1
31	77°47.95′ N 18°56.62′ E	13	91	−1.28	34.9	MW	6.6 ± 0.5	Grayish-brown mud, black and gray soft clay, stones	58.7	37.7	93.2	8.7
28	77°30.56′ N 18°40.06′ E	11	115	−1.8	35.21	BSW	7.0 ± 0.3	Grayish-brown watered clay mud, gray and black clay, gravel	41.2	54.0	90.2	8.8
Transect II												
78(17)	77°20.16′ N 19°34.20′ E	34	151	−1.87	35.37	BSW	7.8 ± 0.8	Brown mud, gray and black clay, shells	56.4 ^a^	30.7 ^a^	80.5 ^a^	–
79	77°05.22′ N 19°36.48′ E	58	130	−1.83	35.29	BSW	8.6 ± 1.0	Sandy mud, gray and black viscous clay	66.3 ^a^	22.0 ^a^	73.1 ^a^	–
80	76°50.16′ N 19°37.08′ E	65	157	−1.25	35.07	MW	9.0 ± 0.9	Brown mud, gray and black soft clay, shells, a small pebbles	69.2 ^a^	20.0 ^a^	71.1 ^a^	–
78(19)	76°46.44′ N 19°41.58′ E	68	149	0.17	34.9	PW	9.2 ± 1.0	Brown sandy mud, gray soft clay, a small fine stones	70.4	18.9	72.1 ^b^	8.9
81	76°35.40′ N 19°36.54′ E	69	218	0.77	34.94	PW	9.4 ± 0.9	Sandy mud, black and gray soft clay, broken shells, a small stones	70.8 ^a^	18.8 ^a^	68.0 ^c^	–

Note: St.—station, CC—coordinates, L—Distance to the shore (km), D—depth (m), T—temperature (°C), S—salinity, IFP—mean duration (±SE) of the ice-free period, WM—water mass, BSW—brine-enriched shelf water, PW—polar front water, MW—modified water, AC—aleurite content (0.1–0.01 mm particles in the 0–2 cm layer, %), PC—pelite content (<0.01 mm particles in the 0–2 cm layer, %), SC—silt content (<0.05 mm particles in the 0–2 cm layer, %), LOI—loss on ignition (% in the 0–2 cm layer). a—calculated values; b—from Meshcheryakov et al. [85]; c—from Lockwood [97].

**Table 2:** Macrozoobenthic characteristics in Storfjord, Svalbard.

St.	α	s	Н′	J′	D_E_	N ± SE	CV_N_	B ± SE	CV_B_	AIM	NF
Shallow transect I											
39	91	60.0	2.9	0.65	−0.24	3923 ± 263	11.5	265 ± 46	29.9	0.07	27
37	135	85.3	3.6	0.74	−0.47	5540 ± 92	3.0	264 ± 102	66.6	0.05	29
34	73	46.7	2.8	0.66	−0.22	3743 ± 248	11.5	192 ± 45	40.9	0.05	25
31	86	56.7	2.9	0.66	−0.25	5470 ± 542	17.2	245 ± 47	33.1	0.04	28
28	108	69.0	3.0	0.65	−0.31	7627 ± 1384	31.4	167 ± 8	8.4	0.02	31
X ± SE	98 ± 11	63.5 ± 6.5	3.0 ± 0.1	0.67 ± 0.02	−0.30 ± 0.04	5260 ± 702	14.9 ± 4.7	226 ± 20	35.8 ± 9.4	0.05 ± 0.01	28 ± 1
Deep transect II											
78(17)	100	63.3	2.8	0.62	−0.13	5660 ± 317	9.7	148 ± 13	14.9	0.03	29
79	109	69.3	3.3	0.71	−0.34	5477 ± 427	13.5	182 ± 36	33.9	0.03	28
80	78	54.0	2.9	0.66	−0.33	5970 ± 122	3.5	146 ± 14	16.7	0.02	28
78(19)	102	67.7	2.9	0.64	−0.18	8527 ± 264	5.4	167 ± 46	48.0	0.02	29
81	122	80.3	3.0	0.63	−0.48	8977 ± 460	8.9	104 ± 12	19.9	0.01	32
X ± SE	102 ± 7	66.9 ± 4.3	3.0 ± 0.1	0.65 ± 0.01	−0.29 ± 0.06	6922 ± 754	8.2 ± 1.7	149 ± 13	26.7 ± 6.2	0.02 ± 0.00	29 ± 1

Note. St.—station, α—alpha diversity (number of species per 0.3 m^2^), s—average number of species per sample, Н′—Shannon index, J′—Pielou evenness, D_E_—difference in evenness index, N—abundance (ind. m^−2^), B—biomass (g m^−2^), CV—coefficient of variation, AIM—average individual mass (g), X—mean, SE—standard error, NF—number of functional groups.

**Table 3:** Frequency of occurrence (FO, %) and abundance (%)/biomass (%) of dominating benthic taxa with FO > 50% in the Storfjord. Stations are arranged in a north–south geographical orientation.

Taxa	FO	Stations									
		Shallow Transect I					Deep Transect II				
		39	37	34	31	28	78(17)	79	80	78(19)	81
*Lumbrineris mixochaeta* (P)	100	15.2/1.7	6.5/0.2	20.8/1.3	15.1/1.2	12.3/1.8	6.7/1.6	4.6/1.3	9.7/3.2	2.2/1.1	1.9/0.9
*Chaetozone setosa* (P)	100	5.7/0.3	2.4/0.1	8.1/0.4	10.3/0.5	9.2/0.7	6.4/0.9	5.1/0.6	10.3/1.4	3.2/0.6	0.4/0.2
*Galathowenia oculata* (P)	100	0.3/0.0	3.7/0.1	1.2/0.0	2.7/0.1	0.6/0.0	6.8/0.4	10.1/0.3	18.0/0.7	23.2/1.1	4.0/0.3
*Heteromastus filiformis* (P)	100	3.5/0.1	0.8/0.0	2.2/0.2	5.6/0.2	2.6/0.1	16.3/0.5	10.8/0.3	10.5/0.4	10.3/0.8	2.5/0.3
*Leitoscoloplos acutus* (P)	100	10.9/0.2	3.0/0.1	13.9/0.3	8.1/0.2	9.0/0.5	6.0/0.5	7.1/1.2	6.6/0.5	4.5/0.4	1.3/0.3
*Maldane sarsi* (P)	100	3.7/10.3	10.3/19.1	3.6/6.7	16.0/33.9	8.3/36.5	6.4/18.1	4.8/6.4	1.9/7.1	4.7/26.8	16.5/5.0
*Eteone flava* (P)	97	1.3/0.0	0.4/0.0	0.9/0.1	1.2/0.1	0.6/1.1	1.1/0.2	0.7/0.1	0.7/0.2	0.8/0.3	0.2/0.0
*Aphelochaeta marioni* (P)	97	6.8/0.3	1.3/0.1	6.3/0.2	3.5/0.2	2.4/0.2	8.8/0.9	2.7/0.2	4.3/0.3	7.7/0.6	1.7/0.2
*Eudorella emarginata* (C)	93	1.2/0.0	0.3/0.0	1.1/0.1	1.0/0.0	0.4/0.1	0.6/0.1	0.4/0.0	0.7/0.1	0.2/0.0	0.0/0.0
*Levinsenia gracilis* (P)	93	2.4/0.0	3.5/0.0	1.2/0.0	5.7/0.1	3.8/0.1	0.1/0.0	2.1/0.0	0.8/0.0	0.7/0.0	0.9/0.0
*Ennucula tenuis* (B)	93	5.7/6.4	0.7/0.1	6.5/8.4	0.2/0.0	0.1/0.0	0.2/0.0	0.9/0.0	0.7/0.0	0.2/0.0	0.1/0.0
*Yoldiella nana* (B)	90	0.3/0.0	0.4/0.0	0.2/0.0	0.3/0.0	1.5/0.1	9.7/0.9	14.5/1.3	2.9/0.3	7.6/0.6	6.8/0.8
*Ophiocten sericeum* (E)	87	0.3/0.2	0.2/1.0	0.2/0.1	0.4/1.1	0.5/1.3	0.5/1.7	2.0/2.8	0.6/0.7	0.2/0.3	0.4/0.2
*Actinocythereis dunelmensis* (C)	83	2.0/0.0	3.0/0.0	4.0/0.0	1.4/0.0	2.3/0.0	0.1/0.0	0.0/0.0	0.2/0.0	2.5/0.0	0.5/0.0
*Terebellides stroemii* (P)	80	0.3/0.0	1.0/0.3	0.1/0.1	0.7/0.2	0.3/0.0	0.9/1.3	0.4/0.3	0.1/1.0	0.0/0.0	0.1/0.0
*Amphiura sundevalli* (E)	80	0.4/0.8	3.4/1.4	1.3/5.4	0.4/2.5	0.3/1.8	0.4/2.1	0.3/0.8	3.8/3.2	0.3/1.7	-
*Lysippe labiata* (P)	80	0.3/0.0	0.7/0.1	0.4/0.1	1.2/0.1	0.6/0.2	0.2/0.0	0.2/0.0	-	0.0/0.0	0.1/0.0
*Diastylis goodsiri* (C)	77	0.3/0.0	0.7/0.5	0.1/0.0	0.5/0.7	0.1/0.0	0.3/2.1	2.0/1.6	0.8/3.4	0.5/1.5	-
*Frigidoalvania janmayeni* (G)	77	0.5/0.0	0.5/0.0	-	-	0.2/0.0	3.9/1.3	3.5/0.8	7.1/1.8	1.8/0.1	0.9/0.4
*Polycirrus arcticus* (P)	70	0.6/0.2	0.1/0.1	1.4/1.0	0.7/0.7	0.1/0.1	2.7/2.4	1.7/1.3	1.2/2.2	-	-
*Caecognathia elongata* (C)	70	0.4/0.0	0.4/0.0	0.1/0.0	0.7/0.0	0.4/0.0	-	0.7/0.0	0.1/0.0	0.6/0.0	0.1/0.0
*Eucratea loricata* (Br)	70	0.2/0.0	0.1/0.0	0.1/0.0	0.1/0.0	0.1/0.7	0.1/0.0	0.1/0.0	0.1/0.0	0.1/0.0	0.1/0.1
*Pholoe assimilis* (P)	67	0.3/0.0	0.4/0.0	0.0/0.0	1.5/0.0	1.1/0.0	0.5/0.0	0.5/0.0	0.5/0.0	-	0.0/0.0
*Philomedes globosus* (C)	67	-	0.8/0.0	0.3/0.0	2.4/0.0	2.5/0.1	-	0.1/0.0	0.1/0.0	0.2/0.0	0.0/0.0
*Tharyx sp.* (P)	63	3.6/0.1	0.6/0.0	1.0/0.0	0.4/0.0	0.3/0.0	1.2/0.1	0.3/0.0	-	-	0.0/0.0
Syllidae g.sp. (P)	60	0.5/0.0	0.5/0.0	0.4/0.0	0.3/0.0	0.5/0.1	0.1/0.0	-	-	0.1/0.0	0.0/0.0
*Heterocyprideis sorbyana* (C)	57	0.7/0.0	13.8/0.0	0.4/0.0	1.3/0.0	10.9/0.1	-	-	-	5.6/0.1	0.0/0.0
Cirratullidae g.sp. (P)	57	0.3/0.0	0.1/0.0	-	0.5/0.0	2.8/0.1	0.5/0.0	0.5/0.0	-	0.5/0.0	0.0/0.0
*Ctenodiscus crispatus* (E)	57	-	0.1/0.0	-	0.2/16.0	0.1/9.8	0.7/40.2	0.5/11.3	0.4/0.3	0.2/13.6	0.2/4.7
*Phyllodoce groenlandica* (P)	53	0.2/0.0	0.1/0.0	-	0.2/0.0	0.3/1.2	-	0.1/0.0	0.2/1.3	0.3/1.6	0.1/0.4
*Heterocyprideis fascis* (C)	53	0.1/0.0	0.2/0.0	1.0/0.0	1.0/0.0	8.1/0.1	0.1/0.0	-	-	0.4/0.0	-
*Nuculana pernula* (B)	53	1.2/1.9	0.8/1.2	0.6/3.7	0.1/0.0	0.3/0.4	0.4/0.1	0.1/0.0	-	-	-
*Mendicula ferruginosa* (B)	50	-	-	-	-	-	0.3/0.0	1.8/0.0	1.1/0.1	2.5/0.1	27.3/1.5
*Spiochaetopterus typicus* (P)	50	-	-	0.2/1.0	0.1/0.0	0.0/1.3	0.1/1.9	0.1/0.6	2.2/39.2	1.1/17.5	3.3/64.0

Note: B—Bivalvia; Br—Bryozoa; C—Crustacea; E—Echinodermata; G—Gastropoda; P—Polychaeta.

**Table 4:** Results of SIMPER analysis: contribution of the first seven benthic species to the overall dissimilarity between clusters.

**Cluster 1–Cluster 2**				**Cluster 1–Cluster 3**			
**Taxon**	**Average Dissimilarity = 54.10%**			**Taxon**	**Average Dissimilarity = 52.66%**		
	**Av. Diss**	**Contrib**	**Cum**		**Av. Diss**	**Contrib**	**Cum**
*Yoldia hyperborea*	3.98	7.40	7.35	*Yoldia hyperborea*	4.17	7.91	7.91
*Myriochele heeri*	3.35	6.20	13.55	*Ennucula tenuis*	3.02	5.73	13.64
*Ennucula tenuis*	3.11	5.70	19.28	*Nothria hyperborea*	2.30	4.36	18.00
*Bathyarca glacialis*	2.23	4.10	23.41	*Ctenodiscus crispatus*	2.28	4.33	22.33
*Tridonta montagui*	1.86	3.40	26.85	*Ciliatocardium ciliatum*	1.70	3.23	25.56
*Ciliatocardium ciliatum*	1.81	3.30	30.20	Nematoda g.sp.	1.68	3.18	28.75
Nematoda g.sp.	1.79	3.30	33.5	*Diastylis goodsiri*	1.66	3.142	31.89
**Cluster 1–Cluster 4**				**Cluster 2–Cluster 3**			
**Taxon**	**Average Dissimilarity = 62.43%**			**Taxon**	**Average Dissimilarity = 49.96%**		
	**Av. Diss**	**Contrib**	**Cum**		**Av. Diss**	**Contrib**	**Cum**
Terebellidae g.sp.	4.84	7.76	7.76	*Myriochele heeri*	2.83	5.66	5.66
*Yoldia hyperborea*	4.54	7.28	15.03	Nematoda g.sp.	2.41	4.82	10.48
*Ennucula tenuis*	3.25	5.20	20.24	*Tridonta montagui*	1.74	3.48	13.96
Nematoda g.sp.	1.94	3.11	23.35	*Ciliatocardium ciliatum*	1.51	3.01	16.97
*Actinocythereis dunelmensis*	1.89	3.02	26.37	*Phyllodoce groenlandica*	1.47	2.93	19.9
*Ciliatocardium ciliatum*	1.85	2.97	29.34	*Nothria hyperborea*	1.36	2.72	22.62
*Nuculana pernula*	1.76	2.83	32.17	*Tridonta borealis*	1.35	2.71	25.33
**Cluster 2–Cluster 4**				**Cluster 3–Cluster 4**			
**Taxon**	**Average Dissimilarity = 55.83%**			**Taxon**	**Average Dissimilarity = 47.32%**		
	**Av. Diss**	**Contrib**	**Cum**		**Av. Diss**	**Contrib**	**Cum**
Terebellidae g.sp.	4.88	8.74	8.74	Terebellidae g.sp.	4.12	8.70	8.70
*Myriochele heeri*	3.16	5.66	14.4	Nematoda g.sp.	2.56	5.41	14.12
*Actinocythereis dunelmensis*	1.85	3.32	17.72	*Actinocythereis dunelmensis*	1.85	3.92	18.03
*Tridonta montagui*	1.84	3.29	21.01	*Nothria hyperborea*	1.50	3.18	21.21
*Phyllodoce groenlandica*	1.62	2.89	23.91	*Ciliatocardium ciliatum*	1.49	3.15	24.36
*Tridonta borealis*	1.17	2.09	26.00	*Scoletoma fragilis*	1.32	2.78	27.14
*Pholoe assimilis*	1.11	1.99	27.99	*Ctenodiscus crispatus*	1.28	2.70	29.84

Note: Av. Diss—average dissimilarity (%), Contrib—contribution (%), Cum—cumulative contribution (%).

**Table 5:** Benthic community types in Storfjord, with the 10 most important species in each type.

**Type 1: *Yoldia hyperborea*** **Stations 34 and 39**				**Type 2: *Maldane sarsi*** **Stations 28, 31, and 37**			
**Dominant Species**	**R**	**N**	**B**	**Dominant species**	**R**	**N**	**B**
*Yoldia hyperborea*	175.9	76.7	57.1	*Maldane sarsi*	394.7	693.3	64.9
*Maldane sarsi*	107.7	138.3	19.9	*Lumbrineris mixochaeta*	99.8	706.7	2.1
*Lumbrineris mixochaeta*	99.8	685	3.3	*Nothria hyperborea*	38.4	20.0	3.0
*Ennucula tenuis*	93.3	233.3	16.5	*Tridonta borealis*	31.9	24.4	36.3
Nemertini g.sp.	69.6	18.3	7.2	*Tridonta montagui*	30.7	73.3	26.0
*Bathyarca glacialis*	61.3	11.7	25.1	*Aglaophamus malmgreni*	17.9	12.2	1.3
*Ciliatocardium ciliatum*	50.8	1.7	37.2	*Ctenodiscus crispatus*	17.6	7.8	18.6
*Scoletoma fragilis*	37.5	3.3	5.2	*Chaetozone setosa*	14.8	466.7	0.9
*Nuculana pernula*	24.5	35.0	6.1	*Eteone flava*	13.6	43.3	0.7
*Thyasira gouldi*	19.0	156.7	2.2	*Aphelochaeta marioni*	5.0	148.9	0.3
**Type 3: *Maldane sarsi* + Nemertini g.sp.** **Stations 78(17) and 79**				**Type 4: *Spiochaetopterus typicus*** **Stations 78(19), 80, and 81**			
**Dominant Species**	**R**	**N**	**B**	**Dominant Species**	**R**	**N**	**B**
*Maldane sarsi*	125.2	311.7	19.2	*Spiochaetopterus typicus*	231.2	174.4	51.1
Nemertini g.sp.	137.4	20.0	15.6	*Maldane sarsi*	130.6	664.4	20.1
*Lumbrineris mixochaeta*	64.6	315.0	2.4	*Lumbrineris mixochaeta*	66.1	312.2	2.5
*Ctenodiscus crispatus*	47.4	33.3	40.0	*Diastylis goodsiri*	26.3	28.9	2.5
*Nepftys ciliata*	47.1	8.3	5.3	*Nepftys paradoxa*	21.7	2.2	2.9
*Ciliatocardium ciliatum*	44.2	6.7	21.6	*Phyllodoce groenlandica*	21.7	13.3	1.7
*Diastylis goodsiri*	34.3	63.3	3.0	*Thracia myopsis*	21.4	2.2	11.1
*Polycirrus arcticus*	24.7	123.3	2.9	*Galathowenia oculata*	20.8	1097.8	1.1
*Chaetozone setosa*	16.1	321.7	1.2	*Chaetozone setosa*	14.5	310.0	1.0
*Aphelochaeta marioni*	12.4	321.7	0.8	*Aphelochaeta marioni*	9.8	357.2	0.6

Note: R—metabolic rate (kJ m^−2^), N—abundance (ind. m^−2^), B—biomass, (g m^−2^).

**Table 6:** List of environmental variables contributed to the RDA models based on abundance of benthic taxa and abundance of functional groups in Stofjord.

Variable	EV	F	P	Variable	EV	F	P
Taxa Abundance				Abundance of Functional Groups			
IFP	59	11.5	0.001	IFP	54	9.35	0.001
T	10	2.35	0.094	T	7	1.32	0.248
De	6	1.5	0.232	De	3	0.93	0.447
Dist	8	2.19	0.073	Dist	10	3.63	0.047
Fine	4	1.21	0.317	Fine	8	1.88	0.181
S	1	0.33	0.816	S	9	1.69	0.21

T—temperature (°C); S—salinity; De—depth (m); Fine—fine sediment content (%); IFP—duration of ice-free period (months); Dist—distance to shore (km)/the degree of siltation; EV—explained variation (%); F—pseudo F-ratio; P—probability level.

## Data Availability

All data are included in the text.

## Appendix A

**Table A1:** List of macrobenthic taxa registered in Storfjord. “+” indicates the presence of a taxon.

Taxon	Station									
	39	37	34	31	28	78(17)	79	80	78(19)	81
Phylum Porifera										
Porifera g.sp.					+	+			+	
Phylum Cnidaria										
Class Hydrozoa										
*Abietinaria pulchra* (Nutting, 1904)	+									
*Eudendrium* sp. 1	+									
*Eudendrium* sp. 2		+								
*Eudendrium* sp. 3		+								
*Halecium curvicaule* Lorenz, 1886		+								
*Lafoea fruticosa* (M. Sars, 1850)	+	+			+					
*Ptychogena crocea* Kramp and Damas, 1925				+						
*Rhizocaulus verticillatus* (L., 1758)					+					
*Sertularia albimaris* Mereschkowsky, 1878						+				+
*Symplectoscyphus tricuspidatus* (Alder, 1856)				+				+		
*Sertularella rugosa* (L., 1758)									+	
Class Antozoa										
*Synarachnactis lloydii* (Gosse, 1859)										+
*Gersemia rubiformis* (Ehrenberg, 1834)	+						+			
*Paraedwardsia arenaria* Carlgren in Nordgaard, 1905	+	+	+				+			+
Phylum Nemertea										
Nemertea g.sp.	+	+	+	+	+	+	+	+	+	+
Phylum Nematoda										
Nematoda g.sp.	+	+	+	+	+	+	+	+	+	+
Phylum Annelida										
Class Polychaeta										
*Aglaophamus malmgreni* (Théel, 1879)	+	+		+	+	+	+			+
*Amblyosyllis finmarchica* (Malmgren, 1867)						+				
*Ampharete finmarchica* (M. Sars, 1865)	+				+		+			
*Ampharete lindstroemi* Malmgren, 1867 sensu Hessle, 1917				+						
Ampharetidae g.sp.	+	+		+	+	+		+	+	
*Amphicteis gunneri* (M. Sars, 1835)							+			
*Amphicteis ninonae* Jirkov, 1985		+								
*Anobothrus gracilis* (Malmgren, 1866)					+	+				
*Aphelochaeta marioni* (Saint-Joseph, 1894)	+	+	+	+	+	+	+	+	+	+
*Aphelochaeta* sp. 1	+	+	+	+	+	+	+	+	+	+
*Aphelochaeta* sp. 2					+					
*Aphelochaeta* sp. 3	+									
*Apistobranchus tullbergi* (Théel, 1879)		+		+	+					
*Aricidea (Strelzovia) quadrilobata* Webster and Benedict, 1887								+	+	+
*Aricidea hartmani* Strelzov, 1968	+	+			+	+	+	+	+	
*Artacama proboscidea* Malmgren, 1866				+						
*Brada strelzovi* Jirkov and Filippova in Jirkov, 2001							+			
*Bradabyssa villosa* (Rathke, 1843)	+	+	+		+	+				
*Bylgides elegans* (Théel, 1879)		+								
*Bylgides groenlandica* (Malmgren, 1867)										+
*Bylgides* sp.							+			+
*Capitella capitata* (Fabricius, 1780)			+		+		+		+	
*Chaetozone setosa* Malmgren, 1867	+	+	+	+	+	+	+	+	+	+
*Chirimia biceps biceps* (Sars, 1861)					+					+
*Chone duneri* Malmgren, 1867							+	+	+	+
*Chone murmanica* Lukasch, 1910				+	+		+	+	+	+
*Chone* sp.						+				
Cirratullidae g.sp.1	+	+		+	+	+	+		+	+
Cirratullidae g.sp. 2					+					
*Cirratulus cirratus* (O.F. Müller, 1776)	+		+	+	+		+			
*Cirrophorus branchiatus* Ehlers, 1908										+
*Cistenides hyperborea* (Malmgren, 1866)	+		+	+	+	+	+			
*Cossura longocirrata* Webster and Benedict, 1887			+	+	+				+	+
*Diplocirrus glaucus* (Malmgren, 1867)	+									
*Dipolydora caulleryi* Mesnil, 1897					+					
*Dipolydora coeca* (Öersted, 1843)		+		+	+					
*Dipolydora socialis* (Schmarda, 1861)				+						
*Dipolydora* sp.	+		+		+	+	+			+
*Enipo torelli* (Malmgren, 1865)		+	+	+		+		+		
*Eteone flava* (Fabricius, 1780)	+	+	+	+	+	+	+	+	+	+
*Eteone spetsbergensis* Malmgren, 1865			+							
*Euchone analis* (Kröyer, 1856)	+							+	+	+
Exogoninae g.sp.					+					
*Galathowenia oculata* Zachs, 1923	+	+	+	+	+	+	+	+	+	+
*Gattyana amondseni* (Malmgren, 1867)	+									
*Gattyana cirrhosa* (Pallas, 1766)			+	+	+					
*Glyphanostomum pallescens* (Théel, 1879)					+				+	+
*Harmothoe imbricata* (Linnaeus, 1767)	+									
*Heteromastus filiformis* (Claparède, 1864)	+	+	+	+	+	+	+	+	+	+
*Lanassa venusta venusta* (Malm, 1874)				+		+				
*Laonice cirrata* (M. Sars, 1851)		+				+				
*Leaena ebranchiata* (M. Sars, 1865)				+	+	+				+
*Laphania boecki* Malmgren, 1865									+	
*Leitoscoloplos acutus* (Verrill, 1873)	+	+	+	+	+	+	+	+	+	+
*Levinsenia gracilis* (Tauber, 1879)	+	+	+	+	+	+	+	+	+	+
*Lumbriclymene minor* Arvidsson, 1906		+			+	+		+	+	+
*Lumbrineris mixochaeta* Oug, 1998	+	+	+	+	+	+	+	+	+	+
*Lysippe labiata* Malmgren, 1865	+	+	+	+	+	+	+		+	+
*Maldane arctica* Detinova, 1985		+								
*Maldane sarsi* Malmgren, 1865	+	+	+	+	+	+	+	+	+	+
Maldanidae g.sp.										+
*Melinna elisabethae* McIntosh, 1914		+		+			+		+	+
*Myriochele heeri* Malmgren, 1867		+		+	+					+
*Nepftys ciliata* (Müller, 1779)		+		+		+	+	+		+
*Nepftys paradoxa* Malm, 1874				+				+	+	
Nephtyidae g.sp.		+								
*Nothria hyperborea* (Hansen, 1878)		+			+	+	+			+
*Ophelina cylindricaudata* (Hansen, 1879)		+								+
Oweniidae g.sp.							+			+
*Paradexiospira (Spirorbides) cancellata* (Fabricius, 1780)		+								
*Paradoneis lyra* (Southern, 1914)					+				+	
*Pholoe assimilis* Örsted, 1845	+	+		+	+	+	+	+		+
*Pholoe* sp.			+						+	
*Phyllodoce groenlandica* Örsted, 1842	+	+		+	+		+	+	+	+
*Polycirrus arcticus* Sars, 1865	+	+	+	+	+	+	+	+		
*Polycirrus medusa* Grube, 1850				+						
Polynoidae g.sp.	+	+			+	+			+	
*Praxillella gracilis* (M. Sars, 1861)		+	+	+	+	+	+	+	+	+
*Praxillella praetermissa* (Malmgren, 1865)						+	+			+
*Prionospio cirrifera* (Wirén, 1883)		+		+					+	+
*Pseudoscalibregma parvum* (Hansen, 1879)										+
*Rhodine gracilior* Tauber, 1879		+		+	+		+	+	+	+
*Saphobranchia hirsuta* (Hansen, 1878)										+
*Saphobranchia longisetosa* (Marenzeller, 1890)							+			
*Scalibregma inflatum* Rathke, 1843	+	+	+			+			+	
*Scoletoma fragilis* (O.F. Müller, 1776)	+			+						
*Sosane wireni* Hessle, 1917										+
*Sphaerodoridium kolchaki* sp. n.		+			+					
*Sphaerodoridium minutum* (Webster & Benedict, 1887)						+				
*Spio limicola* Verrill, 1879					+	+	+			+
*Spiochaetopterus typicus* M. Sars, 1856			+	+	+	+	+	+	+	+
*Spiophanes kroyeri* Grube, 1860				+			+	+	+	+
Spirorbinae g.sp.		+			+					
Syllidae g.sp.	+	+	+	+	+	+			+	+
Terebellidae g.sp.		+	+		+	+				
*Terebellides gracilis* Malm, 1874		+								+
*Terebellides* sp.				+						
*Terebellides stroemii* Sars, 1835	+	+	+	+	+	+	+	+	+	+
*Tharyx* sp. 1	+	+	+	+	+	+	+			+
*Tharyx* sp. 2	+	+		+		+	+		+	+
Phylum Priapulida										
*Priapulus caudatus* Lamarck, 1816	+		+		+	+	+	+	+	
Phylum Sipuncula										
Class Sipunculidea										
*Golfingia (G.) elongata* (Keferstein, 1863)	+			+	+	+			+	
*Golfingia (G.) margaritacea* (M. Sars, 1851)							+			
*Nephasoma (N.) diaphanes diaphanes* (Gerould, 1913)						+	+		+	+
*Nephasoma (N.) eremita* (M. Sars, 1851)							+		+	
*Nephasoma (N.) lilljeborgi* (Danielssen & Koren, 1880)									+	
*Nephasoma (N.) abyssorum* (Koren & Danielssen, 1876)	+	+							+	+
*Phascolion strombus strombus* (Montagu, 1804)		+	+	+	+				+	+
*Golfingia (G.) vulgaris vulgaris* (de Blainville, 1827)				+				+		
Phylum Arthropoda										
Class Malacostraca										
Order Amphipoda										
*Aceroides latipes* (G.O. Sars, 1882)	+					+	+	+		
*Ampelisca eschrichti* Krøyer, 1842				+	+		+			
Amphipoda g.sp.							+			
*Anonyx nugax* (Phipps, 1774)					+	+		+		
*Anonyx* sp.			+							
*Argissa hamatipes* (Norman, 1869)										+
*Arrhis phyllonyx* (M. Sars, 1858)							+	+		
*Bathymedon obtusifrons* (Hansen, 1883)										+
*Byblis* sp.		+								
*Centromedon productus* (Goёs, 1866)		+		+	+	+	+	+		
*Centromedon pumilus* (Liljeborg, 1865)									+	+
*Ericthonius megalops* (G.O. Sars, 1879)						+				
*Gammaropsis melanops* G.O. Sars, 1882							+			+
*Gronella groenlandica* (Hansen, 1888)									+	
*Halirages* sp.										+
*Haploops setosa* Boeck, 1871	+	+		+	+					
*Haploops* sp.			+							
*Haploops tubicola* Liljeborg, 1855	+	+	+		+	+	+	+	+	+
*Hippomedon propinqvus* G.O. Sars, 1890										+
*Idunella aequicornis* (G.O. Sars, 1876)		+		+	+					
*Ischyrocerus megacheir* (Boesk, 1871)						+				
*Ischyrocerus megalops* G.O. Sars, 1894	+				+				+	
*Lepidepecreum umbo* (Goёs,1866)	+		+							
*Menigrates obtusifrons* (Boeck, 1861)						+	+	+		
*Metopa boeckii* G.O. Sars, 1892					+					
*Metopa* sp.										+
*Neohela monstrosa* (Boeck, 1861)		+								
*Onisimus derjugini* Gurjanova, 1929			+		+					
*Onisimus litoralis* (Krøyer, 1845)						+				
*Onisimus normani* (G.O. Sars, 1895)						+				
*Onisimus* sp.							+	+		
*Orchomene* sp.							+			
*Paradulichia typica* Boeck, 1870					+					+
*Paraphoxus oculatus* (G.O. Sars, 1879)										+
*Photis tenuicornis* G.O. Sars, 1882		+			+		+		+	
*Quasimelita formosa* Murdoch, 1865						+				
*Tmetonyx cicada* (Fabricius, 1780)									+	+
*Tryphosella spitzbergensis* Chevreux, 1926										+
*Unciola leucopis* (Krøyer, 1845)				+		+	+	+	+	
Order Cumacea										
*Diastylis goodsiri* (Bell, 1855)	+	+	+	+	+	+	+	+	+	
*Diastylis lepechini* Zimmer, 1926		+		+	+		+	+	+	
*Diastylis rathkei* (Krøyer, 1841)										+
*Diastylis spinulosa* Heller, 1875		+			+				+	
*Ektonodiastylis nimia* (Hansen, 1920)		+								+
*Eudorella emarginata* (Krøyer, 1846)	+	+	+	+	+	+	+	+	+	+
*Eudorella spitzbergensis* Zimmer, 1926					+					
*Leptostylis villosa* G.O. Sars, 1869							+			+
*Leucon acutirostris* G.O. Sars, 1864				+	+	+	+		+	+
*Leucon nasica* (Krøyer, 1841)				+		+	+	+		
*Leucon nasicoides* Lillijeborg, 1855						+				
*Leucon nathorsti* Ohlin, 1901					+					
*Leucon pallidus* G.O. Sars, 1864		+								
*Petalosarsia declivis* (Sars, 1865)									+	
Order Decapoda										
*Pagurus pubescens* (Krøyer, 1838)				+						
Order Isopoda										
*Caecognathia elongata* (Krøyer, 1846)	+	+	+	+	+		+	+	+	+
*Calathura brachiata* (Stimpson, 1854)									+	+
*Desmosoma strombergi* Svavarsson, 1988									+	
*Desmosoma tetarta* (Hessler, 1970)		+								
Desmosomatidae g.sp.		+								
*Munna fabricii* Krøyer, 1846					+		+			
*Munna hanseni* Stappers, 1911										+
Order Tanaidacea										
*Akanthophoreus gracilis* (Krøyer, 1847)		+		+					+	
*Pseudosphyrapus anomalus* (G.O. Sars, 1899)		+								+
*Pseudotanais lilljeborgi* G.O. Sars, 1882		+			+			+	+	+
*Thorkelius latiremis* (Hansen, 1913)										+
*Typhlotanais finmarchicus* G.O. Sars, 1881		+		+						
Class Thecostraca										
*Balanus* sp.		+								
Class Ostracoda										
Order Podocopida										
*Actinocythereis dunelmensis* (Norman, 1865)	+	+	+	+	+	+		+	+	+
*Elofsonella concinna* (Jones, 1857)	+	+			+				+	
*Heterocyprideis fascis* (Brady & Norman, 1889) Hazel, 1968	+	+	+	+	+	+			+	
*Heterocyprideis sorbyana* (Jones, 1857)	+	+	+	+	+				+	+
*Rabilimis mirabilis* (Brady, 1868)		+						+	+	+
*Roundstonia macchesneyi* (Brady & Crosskey, 1871)	+	+							+	
*Sarsicytheridea bradii* (Norman, 1865)		+								
Order Myodocopida										
*Philomedes globosus*		+	+	+	+		+	+	+	+
Class Pycnogonida										
*Nymphon hirtipes* Bell, 1855					+					
Phylum Mollusca										
Class Bivalvia										
*Bathyarca glacialis* (Gray, 1824)	+	+	+			+	+	+	+	+
*Ciliatocardium ciliatum* (Fabricius, 1780)			+	+			+	+		
*Cuspidaria arctica* (M. Sars, 1859)	+					+				+
*Cuspidaria* sp.					+		+	+	+	
*Dacrydium vitreum* (Møller, 1842)		+				+	+	+	+	+
*Ennucula tenuis* (Montagu, 1808)	+	+	+	+	+	+	+	+	+	+
*Hiatella arctica* (L., 1767)		+		+						
*Kurtiella derjugini* (Gorbunov, 1952)		+	+	+						
*Macoma calcarea* (Gmelin, 1791)	+		+							+
*Macoma moesta* (Deshayes, 1855)			+							
*Macoma* sp.						+	+			
*Macoma torelli* (A. S. Jensen, 1905)	+									
*Mendicula ferruginosa* (Forbes, 1844)						+	+	+	+	+
*Musculus niger* (J. E. Gray, 1824)	+	+								
*Musculus* sp.			+		+	+				
*Nuculana pernula* (Müller, 1779)	+	+	+	+	+	+	+			
*Parathyasira equalis* (Verrill et Bush, 1898)						+	+	+		+
*Thracia myopsis* Møller, 1842								+	+	
*Thyasira gouldi* (Philippi, 1845)	+		+						+	
Thyasiridae g.sp.				+	+					
*Tridonta borealis* Schumacher, 1817		+	+				+			
*Tridonta montagui* (Dillwyn, 1817)		+		+	+					
*Yoldia hyperborean* (Gould, 1841)	+	+	+				+			
*Yoldiella frigida* (Torell, 1859)		+			+					
*Yoldiella lenticular* (Møller, 1842)						+	+	+	+	+
*Yoldiella lucida* (Lovén, 1846)								+	+	+
*Yoldiella nana* (M. Sars, 1865)	+	+	+	+	+	+	+	+	+	+
Bivalvia g.sp.		+								
Class Gastropoda										
*Admete viridula* (Fabricius, 1780)										+
*Colus sabini* (Gray, 1824)					+					
*Cylichna alba* (T. Brown, 1827)	+		+					+		
*Cylichnoides scalptus* (Reeve, 1855)						+	+			
*Euspira pallida* (Broderip & G. B. Sowerby I, 1829)									+	
*Frigidoalvania cruenta* (Odhner, 1915)			+							
*Frigidoalvania janmayeni* (Friele, 1878)	+	+			+	+	+	+	+	+
*Hermania scabra* (O. F. Müller, 1784)						+				
*Margarites groenlandicus* (Gmelin, 1791)						+	+	+		
*Propebela rugulata* (Reeve, 1846)			+				+			
*Retusa obtusa* (Montagu, 1803)						+	+	+		
*Siphonodentalium lobatum* (G. B. Sowerby II, 1860)										+
*Turritellopsis stimpsoni* Dall, 1919						+	+			
Class Caudofoveata										
*Chaetoderma nitidulum* Lovén, 1844		+				+				+
Phylum Bryozoa										
Class Gymnolaemata										
*Alcyonidium disciforme* (Smitt, 1878)						+	+			+
*Alcyonidium radicellatum* Kluge, 1946	+					+	+			
*Amphiblestrum solidum* (Packard, 1863)		+								
*Arctonula arctica* (M. Sars, 1851)	+									
*Bugulopsis peachii* (Busk, 1851)					+		+		+	
*Callopora craticula* (Alder, 1856)	+		+			+		+	+	+
*Callopora lineata* (Linnaeus, 1767)		+			+					
*Cauloramphus cymbaeformis* (Hinks, 1877)		+			+					
*Copidozoum smitti* (Kluge, 1946)		+								+
*Celleporella hyalina* (Linnaeus, 1767)					+					+
*Celleporina ventricosa* (Lorenz, 1886)		+								
*Cheilopora sincera* (Smitt, 1868)	+		+			+	+			+
*Cribrilina spitzbergensis* Norman, 1903		+								
*Cystisella saccata* (Busk, 1856)	+	+					+	+		+
*Dendrobeania fruticosa* (Packard, 1863)					+					
*Einhornia arctica* (Borg, 1931)		+		+				+		
*Escharella ventricosa* (Hassall, 1842)	+					+	+			
*Escharopsis lobata* (Lamouroux, 1821)		+								
*Escharoides jacksonii* (Waters, 1900)	+									
*Eucratea loricata* (Linnaeus, 1758)	+	+	+	+	+	+	+	+	+	+
*Hemicyclopora emucronata* (Smitt, 1872)		+								
*Hincksipora spinulifera* (Hincks, 1889)		+								
*Hippoporina harmsworthi* (Waters, 1900)		+								
*Hippoporina reticulatopunctata* (Hincks, 1877)		+	+			+			+	+
*Hippothoa divaricata arctica* Kluge, 1906		+								
*Leieschara subgracilis* (d’Orbigny, 1853)	+	+	+		+		+			
*Microporella arctica* Norman, 1903		+								
*Myriozoella crustacea* (Smitt, 1868)	+						+	+	+	+
*Palmiskenea skenei* (Ellis & Solander, 1786)			+							
*Parasmittina jeffreysi* (Norman, 1876)		+								
*Porella acutirostris* Smitt, 1868										+
*Porella belli* (Dawson, 1859)		+								
*Porella fragilis* Levinsen, 1914	+	+							+	
*Pseudoflustra solida* (Stimpson, 1854)					+					+
*Ragionula rosacea* (Busk, 1856)		+								
*Rhamphostomella hincksi* Nordgaard, 1906		+								
*Rhamphostomella scabra* (O. Fabricius, 1824)						+				
*Schizoporella costata* Kluge, 1962	+									
*Stomachetosella magniporata* (Nordgaard, 1906)	+									
*Stomacrustula cruenta* (Busk, 1854)	+	+								
*Tegella armifera* (Hincks, 1880)									+	
*Tricellaria gracilis* (Van Beneden, 1848)		+							+	+
*Tricellaria ternata* (Ellis & Solander, 1786)					+					
*Turbicellepora canaliculata* (Busk, 1881)										+
*Turbicellepora incrassata* (Lamarck, 1816)		+	+							
Class Stenolaemata										
*Crisia eburneodenticulata* Smitt ms in Busk, 1875	+									+
*Exidmonea atlantica* (Forbes in Johnston, 1847)		+				+				+
*Oncousoecia diastoporides* (Norman, 1869)		+								
*Patinella verrucaria* (Linnaeus, 1758)		+					+			+
*Pleuronea fenestra* (Busk, 1859)									+	
*Tubulipora flabellaris* (O. Fabricius, 1780)										+
*Tubulipora* sp.						+		+		+
*Tubulipora soluta* Kluge, 1946	+	+					+			
Bryozoa g.sp.			+		+					
Phylum Brachiopoda										
*Novocrania anomala* (O. F. Müller, 1776)	+	+		+		+				
Phylum Echinodermata										
Class Asteroidea										
*Ctenodiscus crispatus* (Retzius, 1805)		+		+	+	+	+	+	+	+
Class Holothuroidea										
*Ekmania barthi* (Troschel, 1846)					+					
*Eupyrgus scaber* Lütken, 1857			+	+			+	+	+	
*Myriotrochus rinkii* Steenstrup, 1851	+					+	+	+		+
*Psolus phantapus* Strussenfelt, 1765		+								
Class Ophiuroidea										
*Amphiura sundevalli* (Müller & Troschel, 1842)	+	+	+	+	+	+	+	+	+	
*Ophiacantha bidentata* (Retzius, 1805)	+				+				+	+
*Ophiocten sericeum* (Forbes, 1852)	+	+	+	+	+	+	+	+	+	+
*Ophiura sarsi* Lütken, 1855							+	+	+	+
Ophiuroidea g.sp. juv.				+	+			+	+	
Phylum Hemichordata										
Enteropneusta g.sp.				+				+		
Phylum Chordata										
Class Ascidiacea										
*Cnemidocarpa rhizopus* (Redikorzev, 1907)									+	
